# Vaccine-preventable hospitalisations in adult mental health service users: a population study

**DOI:** 10.1017/S0033291723000776

**Published:** 2023-11

**Authors:** Grant Sara, Patrick Gould, Jackie Curtis, Wendy Chen, Michael Lau, Parashar Ramanuj, David Currow, Philip Burgess

**Affiliations:** 1 Northern Clinical School, Faculty of Medicine and Health, University of Sydney, Sydney, Australia; 2 School of Psychiatry, Faculty of Medicine and Health, University of NSW, Randwick, Australia; 3 System Information and Analytics Branch, NSW Ministry of Health, St Leonards, Australia; 4 TMS Australia, Sydney, Australia; 5 Royal National Orthopaedic Hospital, London, England; 6 Faculty of Health and Behavioural Sciences, University of Wollongong, Wollongong, Australia; 7 School of Population Health, University of Queensland, Brisbane, Australia

**Keywords:** Epidemiology, prevention, vaccination

## Abstract

**Background:**

Vaccine-preventable conditions cause preventable illness and may increase mortality in people living with mental illness. We examined how risks of hospitalisation for a wide range of vaccine-preventable conditions varied by age and sex among mental health (MH) service users.

**Methods:**

Linked population data from New South Wales (NSW), Australia were used to identify vaccine-preventable hospitalisations (VPH) for 19 conditions from 2015 to 2020. Adult MH service users (*n* = 418 915) were compared to other NSW residents using incidence rates standardised for age, sex and socioeconomic status. Secondary analyses examined admissions for COVID-19 to September 2021.

**Results:**

We identified 94 180 VPH of which 41% were influenza, 33% hepatitis B and 10% herpes zoster. MH service users had more VPH admissions [adjusted incidence rate ratio (aIRR) 3.2, 95% CI 3.1–3.3]. Relative risks were highest for hepatitis (aIRR 4.4, 95% CI 4.3–4.6), but elevated for all conditions including COVID-19 (aIRR 2.0, 95% CI 1.9–2.2). MH service users had a mean age of 9 years younger than other NSW residents at first VPH admission, with the largest age gap for vaccine-preventable pneumonias (11–13 years younger). The highest relative risk of VPH was among MH service users aged 45–65.

**Conclusions:**

MH service users have increased risk of hospitalisation for many vaccine-preventable conditions. This may be due to reduced vaccination rates, more severe illness requiring hospitalisation, greater exposure to infectious conditions or other factors. People living with mental illness should be prioritised in vaccination strategies.

## Background

People living with mental ill health experience increased rates of chronic and preventable medical conditions, avoidable hospitalisations and premature mortality (Firth et al., 2019). Vaccine-preventable conditions such as influenza, pneumonia, hepatitis and COVID-19 are likely to contribute to this morbidity and mortality.

People living with mental ill health have low vaccination rates. Studies in clinical cohorts with severe mental illness have found influenza vaccination rates from 25% (Lorenz, Norris, Norton, & Westrick, 2013) to as low as 7% (Young, Dosani, Whisler, & Hwang, 2015), and vaccination rates below 10% for pneumococcal pneumonia, measles and hepatitis (Miles, Williams, Luthy, & Eden, 2020). Among US veterans, those with mental health (MH) conditions are up to 20% less likely to receive vaccination for pneumococcal disease or influenza (Druss, Rosenheck, Desai, & Perlin, 2002). In Israel, vaccination rates against COVID-19 were 25% lower in people with non-affective psychoses than in the broader population (Goldberger et al., 2022).

Rates of vaccine-preventable conditions are also increased in people living with MH conditions. In recent meta-analyses, people with serious mental illness had up to fivefold increases in the prevalence of hepatitis B (Ayano et al., 2018; Hughes, Bassi, Gilbody, Bland, & Martin, 2016), with higher rates in men (Ayano et al., 2018) and in people with a diagnosis of schizophrenia (Lluch & Miller, 2019). Fewer studies have examined other vaccine-preventable conditions. In Taiwan's national claims database, people with a broad range of MH conditions were 30% more likely to be diagnosed with herpes zoster (Yang, Chen, & Lin, 2011), and people diagnosed with bipolar disorder were more than twice as likely to be diagnosed with pneumonia or tuberculosis (Chen et al., 2022). A population study from Israel found that people with any MH condition were less likely to be diagnosed with COVID-19, but this may have reflected lower testing rates (Goldberger et al., 2022).

Few studies have examined hospitalisations or deaths due to vaccine-preventable illnesses in large, representative populations of people with a MH condition. Studies from England, Sweden and Denmark have found a two to three times increased risk of hospitalisation for influenza and pneumonia in MH service users, even when controlling for confounders such as smoking status (Crump, Sundquist, Winkleby, & Sundquist, 2013; Davydow et al., 2016; Seminog & Goldacre, 2013). In Israel, people with MH diagnoses had more than twice the population risk of hospitalisation or death after diagnosis with COVID-19 (Goldberger et al., 2022). Two Australian studies examined sub-national (state) populations, finding a two- to fourfold increased risk of hospitalisation for vaccine-preventable conditions including influenza, pneumonia and hepatitis (Mai, Holman, Sanfilippo, & Emery, 2011; Sara et al., 2021).

More evidence about vaccine-preventable hospitalisations (VPH) is required to understand the scale of this issue and to identify priority areas or groups for action. Most studies have focused on hospitalisations for respiratory illness. No study has reported on a broad range of specific vaccine-preventable conditions or presented age-specific risks. Many of the studies above include data from the 1960s to the early 2000s; however, we need updated data because of new vaccine approaches and new patterns of illness, including COVID-19.

The current study describes hospitalisation rates for vaccine-preventable conditions in the state of New South Wales (NSW), Australia. We examine hospitalisation rates over 5 years for 19 specific vaccine-preventable conditions. We compare rates for the overall adult population to those in MH service users, and report age- and sex-specific rates adjusting for differences in socio-economic disadvantage.

## Methods

### Study design

All VPH were identified over 5 years in the state of NSW, Australia using retrospective observational methods. The outcome of interest was the number of VPH (episodes of hospital care) and the number of VPH-related hospital days, expressed as rates per 100 000 population per year.

### Data linkage

Data from NSW public hospitals, private hospitals, public community MH services and the NSW Register of Births Deaths and Marriages were linked by the NSW Centre for Health Record Linkage (CHeReL), using probabilistic record linkage based on individuals' names, date of birth, addresses and health service identifiers. The linkage process is designed to give a false-positive linkage rate of around 5 per 1000 records. More detail on the datasets and linkage methods is provided elsewhere (Sara et al., 2019).

### Study setting

VPH to NSW private and government-funded (public) hospitals were examined. Around 60% of Australian hospital care is provided through state operated public hospitals (Australian Institute of Health & Welfare, 2022a, December 7). Private hospitals mainly provide non-emergency care for individuals opting-in to private health insurance. Hospital diagnoses are recorded using the International Classification of Diseases, 10th Edition, Australian Modification (National Centre for Classification in Health, 2010).

MH service users were defined by receiving any MH care from an NSW public or private hospital, or a state government operated (public) community MH services. Public community MH services provide care to around 2% of the NSW population each year (Australian Institute of Health and Welfare, 2022b, December 12), focusing on emergency care and longer term care of severe or complex disorders. Primary and private MH outpatient services see around 11% of the NSW population each year (Australian Institute of Health and Welfare, 2022b, December 12), and focus mainly on higher prevalence MH conditions: data for those services are not included in the current linkage.

### Primary outcome: vaccine-preventable hospitalisations

The main outcome was admission to any NSW public or private hospital with a primary or secondary diagnosis of a VPH during a 5-year period from 1 July 2015 to 30 June 2020. We examined 19 vaccine-preventable conditions, using specifications of the Australian Commission on Quality and Safety in Health Care (Falster & Jorm, 2017) supplemented with additional codes. These were divided into three subgroups: (i) respiratory conditions (influenza, pneumococcal pneumonia, haemophilus pneumonia, pertussis, diphtheria, COVID-19); (ii) hepatitis B and (iii) other vaccine-preventable conditions (herpes zoster, tuberculosis, varicella, rotaviral enteritis, mumps, measles, haemophilus meningitis, tetanus, rubella, cholera, acute poliomyelitis). Hepatitis B was grouped separately because of its high prevalence and because its mode of transmission and associations were assumed to be different from those of other conditions. Hepatitis C was not included as it is not currently vaccine preventable.

### Mental health cohort definition

MH service users were defined as NSW residents aged 18–100 years who had any MH hospitalisation or public community MH contact between 1 July 2013 and 30 June 2020. MH hospitalisations were defined as episodes of care in a public or private hospital with a primary diagnosis of a non-organic MH condition (ICD-10 codes F10-F99) or at least one day in a designated MH unit. Community contacts included face-to-face or telehealth contacts with NSW public community MH services. Services to non-NSW residents, administrative contacts, case conferences and contacts by community teams with hospital inpatients were excluded. Sex and area of residence were defined at the first (index) contact in the observation period. Age was estimated at the midpoint of the study period. MH status was treated as time-dependent, with events (hospitalisations) and exposure time (person years) calculated from the date of each person's index MH contact in the study period, or from the start of the study period (1 July 2015) if the index contact occurred during the 2-year pre-study look-back period. Deaths during the study period were identified by linkage to the NSW Register of Births, Deaths and Marriages. If death occurred during the study period, time from the date of death to the study end was excluded when calculating hospitalisation rates.

### Data analysis

Data assembly and standardisation were conducted in SAS Enterprise Guide v7.15. VPH rates per 100 000 person-years were calculated for MH and non-MH groups. VPH episode and day rates were calculated for (i) any VPH, (ii) three VPH groups (respiratory, hepatitis B, other) and (iii) 19 individual VPH conditions. Episodes with multiple VPH diagnoses were counted separately for condition-specific rates but treated as a single episode when calculating overall VPH rates.

For calculation of standardised rates, admission rates were first calculated separately for each study group, subgroup, vaccine preventable condition group, individual vaccine preventable condition and stratum of age (18–24, 25–34, 35–44, 45–54, 55–64, 65–75, 75–84, 85+), sex and quintile of socioeconomic disadvantage. Socioeconomic disadvantage was estimated from the person's area of residence, using the Australian Bureau of Statistics Index of Relative Socioeconomic Disadvantage (IRSD) (Australian Bureau of Statistics, 2006). This index is calculated for Australian geographical areas using 17 census-derived variables measuring income, government welfare support, education, home ownership, employment, household structure and English language proficiency. Denominators for rate calculations were (i) for MH service users: stratum-specific populations from the MH cohort and (ii) for other NSW residents: census-derived population estimates from the Australian Bureau of Statistics for the midpoint of the study period, after subtracting the relevant MH service user population. Adjusted incidence rate ratios (aIRRs) and 95% log normal confidence intervals were then calculated by direct standardisation for age, sex and socioeconomic status, using the SAS procedure ‘Proc STDRATE’ (SAS Institute Inc, 2020, October 28). Standardised rates were not calculated where the number of VPH events in the MH cohort was less than 20.

A supplementary analysis was conducted to examine admission rates for COVID-19, using public hospital data from July 2020 to September 2021. The original planned analysis included all NSW public and private hospitals; however, private hospital data were available only to June 2020. That covered 3 months of the first wave of the NSW pandemic (April to June 2020), during which hospital admissions for COVID-19 were uncommon. Public hospital data were also available to September 2021, which included the peak of the much larger NSW COVID-19 Delta wave. Rates and adjusted rate ratios were calculated using the same methods and population denominators as the main analyses.

Two subgroup analyses were conducted. First, we examined VPH rates in MH service users with severe or persistent mental illness (SPMI), who were defined as people with (i) any diagnosis of schizophrenia, schizoaffective disorder, bipolar disorder or psychotic depression, or (ii) more than two years of contact with MH services, as measured from the dates of their first and last MH service contacts during the observation and pre-study lookback periods. Second, we examined VPH rates in MH service users who had received hospital care, compared to people whose only MH service contact was with community-based, non-admitted MH services.

### Ethics approval

The study was approved by the NSW Population and Health Service Research Ethics Committee (HREC/17/CIPHS/48. CINSW Refs 2017/|HRE1105, 2019/UMB0208), and the Aboriginal Health and Medical Research Council of NSW (Ref 1564/19).

## Results

We identified 500 548 people who had at least one in-scope contact with NSW MH services in the study or look-back periods. Of these, we excluded 62 514 (12.5%) people aged under 18 at first contact, 12 987 (2.6%) with no valid age recorded, and 72 (0.01%) with sex missing or recorded as ‘other than male or female’. We also excluded 5706 people (1.1%) who had contact in the pre-study look-back period but died before the start of the observation period. After these exclusions 418 915 MH service users were included in calculation of standardised rates. Nineteen percent had a diagnosis of a psychotic disorder recorded, and approximately half (51%) received community MH care only (see online Supplementary Table S1). Compared to NSW population estimates for the mid-point of the study period (Jan 2018), MH service users were more likely to be female, aged under 45, and to live in regions in the most disadvantaged two quintiles of the NSW population (Table 1).

**Table 1.** Cohort description

	MH service users	NSW population aged 18+
	*n* (% of group)	*n* (% of group)
Total	418 915 (100%)	6 162 602 (100%)
Sex		
Female	217 843 (52%)	3 137 557 (51%)
Male	201 072 (48%)	3 025 046 (49%)
Age group		
18–24	70 920 (17%)	733 781 (12%)
25–34	87 513 (21%)	1 186 254 (19%)
35–44	80 886 (19%)	1 050 450 (17%)
45–54	66 529 (16%)	1 001 947 (16%)
55–64	46 695 (11%)	923 220 (15%)
65–74	30 765 (7%)	708 857 (12%)
75–84	21 244 (5%)	387 496 (6%)
85+	14 363 (3%)	170 598 (3%)
Disadvantage quintile		
Q1 Most	70 165 (17%)	1 124 329 (18%)
Q2	116 543 (28%)	1 078 653 (18%)
Q3	76 946 (18%)	1 305 848 (21%)
Q4	68 601 (16%)	1 240 146 (20%)
Q5 Least	71 433 (17%)	1 413 626 (23%)
Unknown	15 227 (4%)	–

*Notes*: Mental health service users aged 18–100, and NSW adult population, July 2015 to June 2020. Estimated NSW adult population at study midpoint (January 2018). Disadvantage quintiles based on person's address of residence, using Index of Relative Socioeconomic Disadvantage (IRSD).

During the study period there were 94 180 VPH to NSW public or private hospitals (Table 2). Nearly all (89%) occurred in public hospitals. These hospitalisations included 97 910 individual vaccine-preventable condition diagnoses, because a small number of admissions included more than one such diagnosis. The most frequently diagnosed individual conditions were influenza (41% of all VPH diagnoses), hepatitis B (33%) and herpes zoster (10%). These three conditions, along with two vaccine-preventable pneumonias (haemophilus pneumoniae, streptococcus pneumoniae) made up 98% of all VPH diagnoses. The study period for the primary analysis included only the first three months of the COVID-19 pandemic; 668 COVID admissions were recorded, including 27 in MH service users. Conditions with fewer than 20 admissions in the MH cohort are not reported separately but have been included in group and state totals. These include mumps (109 total admissions), measles (30), haemophilus meningitis (24), tetanus (16), rubella (12), cholera (10), diphtheria (9) and acute poliomyelitis (9).

**Table 2.** Vaccine-preventable hospitalisations (VPH) of adults (aged 18–100) to NSW public and private hospitals, July 2015 to June 2020, comparing people receiving any hospital or community mental health care to the rest of the NSW population

	VPH episodes (*n*)		Crude rate (per 100k person years)			Adjusted incidence rate ratios (aIRR), (95% CI)	
	MH care	No MH care	MH care	No MH care	IRR	1. Age, sex	2. Age, sex, disadvantage
**Any vaccine-preventable**	**10 679**	**83 501**	**726.7**	**284.6**	**2.6**	**3.1** (**3.0–3.1)**	**3.2** (**3.1–3.3)**
**Hepatitis B**	**4901**	**26 371**	**333**.**5**	**89**.**9**	**3.7**	**4.2** (**4.1–4.4)**	**4.4** (**4.3–4.6)**
**Respiratory infections (1)**	**4918**	**45 820**	**334.7**	**156.1**	**2.1**	**2.7** (**2.6–2.7)**	**2.8** (**2.7–2.9)**
Influenza	3609	35 381	245.6	120.6	2.0	2.5 (2.5–2.6)	2.7 (2.6–2.8)
Haemophilus pneumonia	505	4618	34.4	15.7	2.2	2.7 (2.5–3.0)	2.8 (2.6–3.1)
Streptococcal pneumonia	901	6220	61.3	21.2	2.9	3.5 (3.2–3.7)	3.6 (3.3–3.9)
COVID	27	641	1.8	2.2	0.8	1.0 (0.6–1.4)	0.9 (0.6–1.3)
Pertussis (whooping cough)	55	449	3.7	1.5	2.4	3.0 (2.2–4.0)	3.1 (2.3–4.2)
**Other (2)**	**1029**	**12 535**	**70.0**	**42.7**	**1.6**	**2.1** (**2.0–2.2)**	**2.2** (**2.0–2.3)**
Herpes zoster	761	9106	51.8	31.0	1.7	2.2 (2.0–2.4)	2.3 (2.1–2.5)
Tuberculosis	162	2301	11.0	7.8	1.4	1.6 (1.4–1.9)	1.8 (1.5–2.1)
Varicella (chicken pox)	101	1108	6.9	3.8	1.8	2.2 (1.8–2.7)	2.1 (1.7–2.7)
Rotaviral enteritis	39	401	2.7	1.4	1.9	2.4 (1.7–3.3)	2.5 (1.7–3.5)

VPH, vaccine-preventable hospitalisation; MH, mental health.

Disadvantage measured using index of relative socioeconomic disadvantage (IRSD) of address of residence. (1) Respiratory group total includes nine hospitalisations for diphtheria. (2) Other group total includes 22 hospitalisations for mumps, measles, haemophilus meningitis, tetanus, rubella, cholera, acute poliomyelitis. The sum of individual conditions may exceed group totals because some admissions have more than one VPH diagnosis recorded.

Adjusted incidence rate ratios (aIRR) after standardisation by (1) age and sex, (2) age, sex and socioeconomic disadvantage.

Compared to other NSW residents, MH service users were 2.6 times more likely to experience a VPH, with an aIRR of 3.2 (95% CI 2.1–2.2) after standardising for age, sex and socioeconomic disadvantage. Relative risk was increased for all VPH types other than COVID-19, with the highest relative risk for hepatitis B (aIRR 4.4, 95% CI 4.3–4.6).

A supplementary analysis was conducted for the period from July 2020 to September 2021 to examine hospitalisation rates for COVID, using data for NSW public hospitals only (Table 3). During this period there were 10 186 admissions for COVID-19 to NSW public hospital and ‘hospital in the home’ services (1243 in MH service users, 8943 in other NSW residents). Overall VPH admission rates fell during this period, dropping by 21% in the broader population and 12% in MH service users, with substantial reductions in admission for most VPH types and almost complete absence of influenza hospitalisations. In this period, MH users were twice as likely to have a hospital admission with a COVID-19 diagnosis (aIRR 2.0, 95% CI 1.9–2.2).

**Table 3.** VPH admission rates, NSW public hospitals before and during COVID-19, by VPH condition and MH service user group

PPH condition	VPH episodes (*n*)		Rate (per 100k person years)			Adjusted rate ratio
	MH service users	Other NSW residents	MH service users	Other NSW residents	IRR	aIRR (95% CI)
July 2015 to June 2020						
**Total vaccine-preventable**	**10 263**	**73 370**	**698.4**	**250.0**	**2.8**	**3.3 (3.3–3.4)**
**Hepatitis B**	**4742**	**20 807**	**322.7**	**70.9**	**4.6**	**5.2 (5.0–5.3)**
**Respiratory infections**	**4731**	**42 551**	**322.0**	**145.0**	**2.2**	**2.7 (2.7–2.8)**
Influenza	3456	32 742	235.2	111.6	2.1	2.6 (2.5–2.7)
Haemophilus pneumonia	487	4280	33.1	14.6	2.3	2.8 (2.6–3.1)
Streptococcus pneumoniae	890	5922	60.6	20.2	3.0	3.6 (3.3–3.9)
COVID	26	628	2.2	2.1	1.0	1.1 (0.8–1.7)
**Other**	**957**	**11 195**	**65.1**	**38.2**	**1.7**	**2.2 (2.0–2.3)**
Herpes zoster	709	7927	48.3	27.0	1.8	2.4 (2.2–2.6)
Tuberculosis	153	2228	10.4	7.6	1.4	1.6 (1.3–1.9)
Varicella (chicken pox)	98	1039	6.7	3.5	1.9	2.3 (1.8–2.8)
July 2020 to September 2021 (includes COVID Delta wave)						
**Total vaccine-preventable**	**2662**	**16 556**	**550.7**	**221.2**	**2.5**	**2.7 (2.6–2.8)**
**Hepatitis B**	**988**	**4495**	**204.4**	**60.1**	**3.4**	**3.9 (3.7–4.2)**
**Respiratory infections**	**1466**	**10 030**	**303.3**	**134.0**	**2.3**	**2.2 (2.1–2.4)**
Influenza	23	150	4.8	2.0	2.4	3.2 (2.0–5.1)
Haemophilus pneumonia	68	397	14.1	5.3	2.7	3.4 (2.6–4.5)
Streptococcus pneumoniae	155	642	32.1	8.6	3.7	4.3 (3.6–5.1)
COVID	1243	8943	257.1	119.5	2.2	2.0 (1.9–2.2)
**Other**	**265**	**2365**	**54.8**	**31.6**	**1.7**	**2.4 (2.1–2.7)**
Herpes zoster	218	1786	45.1	23.9	1.9	2.7 (2.4–3.2)
Tuberculosis	30	432	6.2	5.8	1.1	1.1 (0.8–1.7)
Varicella (chicken pox)	50	282	10.3	3.8	2.7	3.4 (2.5–4.7)

**Fig. 1.**
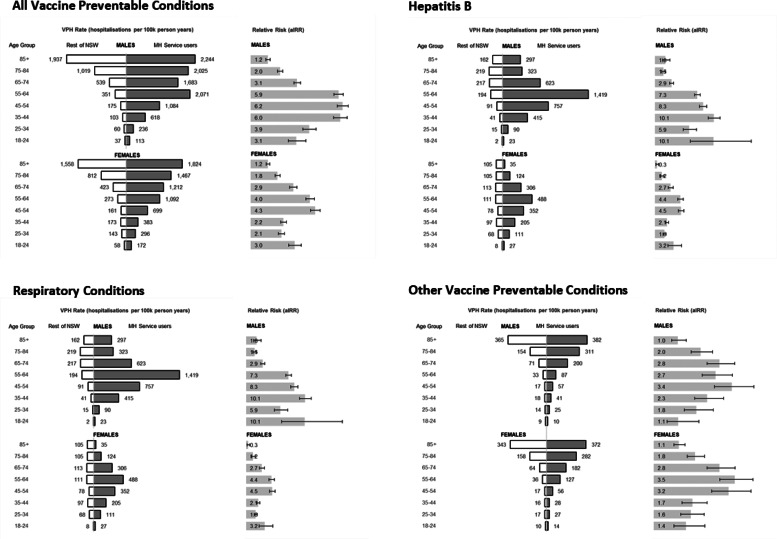
Vaccine-preventable hospitalisations (VPH) in NSW adults aged 18–100, by age group, gender and type of vaccine-preventable condition.

Patterns of VPH differed by age, sex and type of vaccine-preventable condition (Fig. 1 and online Supplementary Table S2). In the broader NSW population VPH rates approximately doubled for each decade over age 55. In MH service users, rates increased from a younger age and the highest relative risks occurred in MH service users aged 35–64. MH service users aged 35–54 had very high relative risks of admission for hepatitis B. In MH service users aged 75 and above, relative risks converged towards population rates.

**Fig. 2.**
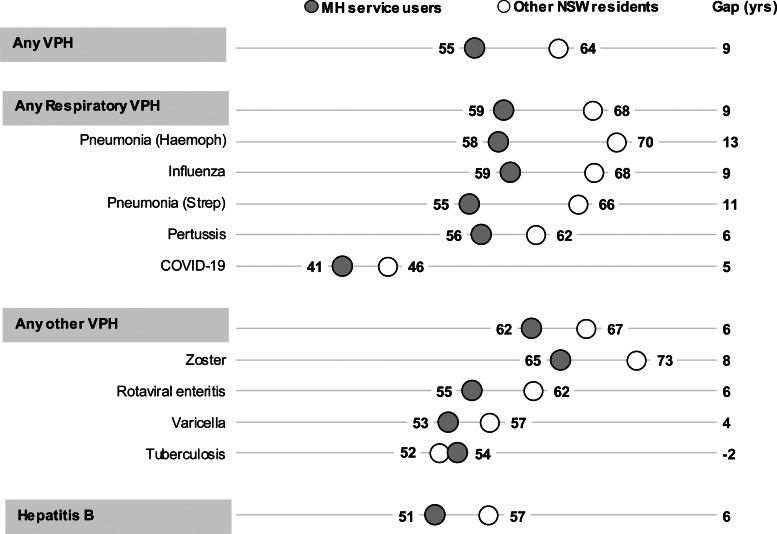
Average age at vaccine-preventable hospitalisation (VPH), comparing mental health (MH) service users to other NSW residents.

Earlier age-related increases in risk of VPH admission were also reflected in younger average age at admission for MH service users. On average, MH service users were 9 years younger than other NSW residents when admitted with a VPH diagnosis (Fig. 2). This age gap was largest for respiratory conditions, particularly for pneumonia due to haemophilus (13 years) or streptococcus (11 years).

Examining the secondary outcome of hospital bed days, MH service users had a slightly longer average length of hospital stay for vaccine-preventable conditions (MH service users 9.7 days, other NSW residents 8.1 days). Because of the combination of a longer length of stay and a higher hospitalisation rate, MH service users experienced 3.8 times more hospital bed days per capita than other NSW residents (95% CI 3.77–3.82) after adjusting for age, sex and socioeconomic status (online Supplementary Table S3).

In subgroup analysis, people with SPMI made up 33% of people and 40% of person-years in the MH cohort (online Supplementary Table S4). Standardised VPH rates were highest in the SPMI group (aIRR 4.0, 95% CI 3.9–4.1), but also significantly elevated in other MH service users (aIRR 2.8, 95% CI 2.8–2.9) compared to other NSW residents. The highest relative risk in the SPMI group was for hepatitis B (aIRR 6.1, 96% CI 5.8–6.3). Both groups of MH service users had significantly increased risk of hospitalisation for all disorders examined. The SPMI subgroup had significantly higher standardised VPH rates than the other MH subgroup overall, for hepatitis and respiratory conditions. However, the other MH subgroup had higher standardised VPH rates than the SPMI subgroup for other vaccine-preventable conditions, including herpes zoster, tuberculosis and varicella. A similar pattern was seen in subgroup analysis based on the type of MH care received (online Supplementary Table S5). Compared to non-MH service users, the relative risk of any VPH was slightly higher in people with any MH hospital care (aIRR 3.6, 95% CI 3.5–3.7) than in people receiving community MH care only (aIRR 3.1, 95% CI 3.0–3.2). Compared to people with community MH care only, people with admitted MH care had a slightly higher rate of VPH for hepatitis and respiratory conditions but slightly lower rate for other vaccine-preventable conditions.

## Discussion

We compared hospitalisation rates for 19 individual vaccine-preventable conditions in 418 915 adult MH service users to a broader population of 6.2 million adults over a 5-year period. Each year in NSW there were nearly 20 000 VPH, occupying more than 120 000 bed days. After adjusting for differences in age, sex and socioeconomic disadvantage, MH service users were 3.2 times more likely than other NSW residents to be admitted to hospital for a vaccine-preventable condition and had 3.8 times more days in hospital per capita for those conditions. The highest relative risk in MH service users was for hepatitis B, but VPH rates were increased across the spectrum of vaccine-preventable conditions, including COVID-19. The rate of VPH was slightly higher in the subgroup of MH service users with severe and persistent mental illness, but other MH service users also had significantly increased rates.

Previous studies have found that people with prior MH service contact were two to three times more likely to be admitted for influenza and pneumonia (Crump et al., 2013; Mai et al., 2011; Seminog & Goldacre, 2013). We found a 2.8-fold increase in admission rate for respiratory conditions, similar to findings of Crump (Crump et al., 2013) and Davydow (Davydow et al., 2016). We also found similar increases for other respiratory conditions including pertussis, as well as for herpes zoster and varicella. Our main analysis covered only the first few months of the COVID-19 pandemic in NSW and did not show increased hospitalisation for COVID-19. However, secondary analysis of a longer period of public hospital data found a twofold risk of COVID admission in MH service users, consistent with recent findings from Israel (Goldberger et al., 2022). These findings of increased hospitalisation rates across a broad spectrum of conditions suggest that causes may also be broad, and that prevention strategies may need to consider diverse conditions and risk groups.

We found that the normal age-related risk curve for VPH appears to be shifted to the left in MH service users. Service users were, on average, almost a decade younger when admitted for vaccine-preventable conditions. MH service users in their 40s and 50s had particularly elevated risk of admission for vaccine-preventable respiratory conditions. For effective prevention, public health strategies may need to consider the earlier onset of chronic medical illness in this group (Firth et al., 2019). In Australia, as in many countries, MH conditions are not currently considered amongst criteria for subsidised access to vaccination at a younger age than the general public for conditions such as influenza, pneumococcal pneumonia or herpes zoster (Australian Technical Advisory Group on Immunisation, 2023). Hepatitis B vaccines are only subsidised for those under 20 years old or for refugee backgrounds. Influenza vaccines are subsidised for those over 65 years old, Aboriginal and Torres Strait Islander people (Australian Technical Advisory Group on Immunisation, 2023), and people with selected medical conditions. Severe mental illness is not included in the list of medical conditions allowing subsidised vaccine access (Australian Technical Advisory Group on Immunisation, 2023). This creates a significant barrier to service providers and individuals in increasing adult vaccination for this population. By contrast, in New Zealand free access to influenza vaccines has been expanded to include people living with a severe mental illness or accessing a MH service (Manatu Hauora New Zealand Ministry of Health, 2023).

Our findings suggest that vaccine-preventable illnesses cause significant harm in people living with MH conditions, and that increased hospitalisation risks are not simply explained by differences in age, sex or socioeconomic status. More study is needed to demonstrate the mechanisms of increased VPH in this group. We do not currently have data on vaccination status to explore these interactions in our study population.

We speculate that increased hospitalisations arise through interactions between increased exposure, reduced vaccination, more severe illness, greater medical comorbidity and other risk factors. Therefore, improving vaccination rates is likely to be an important but not sufficient strategy for reducing vaccine-preventable harms in MH service users. We need strategies to reduce policy barriers, increase access and improve vaccination coverage. We also need to understand and address the many other risk factors likely to also contribute to the development of more severe vaccine-preventable conditions in this group.

Increased rates of hepatitis B in people with severe mental illness may reflect greater exposure through injecting drug use (Hughes et al., 2016; Lluch & Miller, 2019), and for many people exposure to hepatitis B may have occurred prior to the development of mental illness. Additionally, this group may also have greater exposure to respiratory infections due to housing conditions or exposures in health-care settings. Low vaccination rates are likely to contribute to high rates of admission for respiratory conditions (Druss et al., 2002; Miles et al., 2020), but no studies have reported both vaccination rates and harms in the same individuals. People living with MH conditions may have other risk factors and comorbidities causing more severe illness once exposed to infection (Firth et al., 2019). For example, smoking rates are increased in people living with MH conditions, and smoking is a risk factor for more severe illness and ICU admission following respiratory infections, including COVID (Vardavas & Nikitara, 2020). However, some studies have found that increased hospitalisation risk for respiratory illness persists after adjusting for smoking status and comorbid medical or substance use conditions (Crump et al., 2013; Davydow et al., 2016; Mai et al., 2011), suggesting that other factors also contribute. The longer length of stay for VPH admissions in our study may reflect more severe illness, social or other barriers to discharge, or a combination of these factors. There has also been speculation that psychological distress or depressive symptoms may reduce the immune response to vaccination, increasing the risk of illness even when individuals are vaccinated (Abdeljaber, Nair, Schork, & Schwartz, 1994; Ford et al., 2019; Glaser, Robles, Sheridan, Malarkey, & Kiecolt-Glaser, 2003; Segerstrom, Hardy, Evans, & Greenberg, 2012; Wang et al., 2016).

## Strengths and limitations

This study identifies all vaccine-preventable hospital admissions for a population of more than 6 million adults and examines a population-wide cohort of people with MH conditions. This allows a whole population view of a wide range of specific vaccine-preventable conditions. However, there are several limitations to the datasets used in this study.

First, we do not currently have linked data on vaccination status, and so cannot examine the relationship between vaccination status and increased hospitalisation rates.

Second, our dataset does not include community MH service contacts with primary care or private MH providers. Our cohort was defined using hospital and public community MH data and is likely to represent people with more severe or longstanding illness sufficient to require hospital admission or be treated by public community services. They may not be representative of broader groups receiving care only in primary care or private settings for conditions such as depression or anxiety.

Third, our dataset does not include medication prescription data, and we were therefore unable to examine whether psychotropic medications were associated with vaccine preventable hospitalisations. A recent meta-analysis found that exposure to a wide range of psychotropics was associated with increased COVID-related mortality (Vai et al., 2021). However, this may reflect risk associated with the conditions for which those medications were prescribed, rather than independent effects of the medications. Studies controlling for clinical and demographic confounders have reported complex interactions between medications and COVID susceptibility or severity. In people with severe mental illness mood stabilisers were associated with increased risk of COVID infection, while Clozapine and Paliperidone were associated with reduced risk. In a large emergency and acute care cohort (Oskotsky et al., 2021), prior prescription of a selective serotonin reuptake inhibitor was associated with reduced risk of COVID mortality after adjusting for demographic, clinical, co-morbidity and treatment setting variables. More study is needed to understand possible harmful or protective effects of psychotropic medications on vaccine-preventable illnesses (Nemani et al., 2021).

Fourth, community MH records in our large administrative dataset often lack diagnostic information. We therefore combined diagnosis and duration of service contact to define a subgroup with SPMI. While this is a common approach in operational definitions of serious mental illness (Gonzales et al., 2022), it causes imprecision in our subgroup analyses because many people not meeting the operational definition of severe and persistent mental illness may still have had significant risk or impairment.

Finally, our current dataset does not cover the significant COVID-19 Omicron variant wave, which in NSW peaked in the last quarter of 2021 and early 2022. Within the NSW health system, models of care and patterns of hospitalisation for COVID continue to evolve. We plan to revise our current estimates for COVID as further data become available in future linkage cycles.

## Conclusions

COVID-19 reminds us that vaccine programmes are essential in reducing the impact of preventable infections on individuals and health systems. There have been recent calls to ensure that people living with mental illness are prioritised in the development of COVID vaccination strategies (De Hert, Mazereel, Detraux, & Van Assche, 2021) and have equitable access to all vaccination programmes (Equally Well Alliance, 2021, October 1).

We need ongoing study of the mechanisms and impacts of vaccine-preventable harms in people living with mental illness, to identify priority conditions and groups, and to evaluate effective models for intervention.

## Supporting information

Sara et al. supplementary material Sara et al. supplementary material

## Supplementary material

The supplementary material for this article can be found at https://doi.org/10.1017/S0033291723000776.
