# Supplementary material for: Vaccine-preventable hospitalisations in adult mental health service users: a population study
Source: Psychol Med. 2023 Apr 5;53(15):7232–41. doi: 10.1017/S0033291723000776 (PMC10719683; doi:10.1017/S0033291723000776)
Supplement: Sara et al. supplementary material [file S0033291723000776sup001.pdf]

Psychological Medicine

Vaccine-preventable hospitalisations in adult mental health service users: a population study.

--Manuscript Draft--

**Manuscript Number:**	PSM-D-22-01657R2
**Full Title:**	Vaccine-preventable hospitalisations in adult mental health service users: a population study.
**Article Type:**	Original Article
**Corresponding Author:**	Grant Evan Sara, MB.BS. MM. MM. PhD<br>The University of Sydney<br>AUSTRALIA
**Corresponding Author Secondary Information:**	
**Corresponding Author's Institution:**	The University of Sydney
**Corresponding Author's Secondary Institution:**	
**First Author:**	Grant Evan Sara, MB.BS. MM. MM. PhD
**First Author Secondary Information:**	
**Order of Authors:**	Grant Evan Sara, MB.BS. MM. MM. PhD
	Patrick Gould, BN(Hon),MCN
	Jackie Curtis, MB BS, FRANZCP
	Wendy Chen
	Michael Lau, MBChB, MMed, FRANZCP
	Parashar Pravin Ramanuj, MBBS, BSc, MRCPsych
	David C Currow, BMed, MPH, PhD
	Phillip Michael Burgess, MA PhD
**Order of Authors Secondary Information:**	
**Manuscript Region of Origin:**	AUSTRALIA
**Abstract:**	Background<br>Vaccine-preventable conditions cause preventable illness and may increase mortality in people living with mental illness. We examined how risks of hospitalisation for a wide range of vaccine-preventable conditions varied by age and sex among mental health service users.<br>Methods<br>Linked population data from New South Wales (NSW), Australia were used to identify vaccine-preventable hospitalisations (VPH) for 19 conditions from 2015 to 2020. Adult mental health (MH) service users (n=418,915) were compared to other NSW residents using incidence rates standardised for age, sex and socioeconomic status. Secondary analyses examined admissions for COVID-19 to Sept 2021.<br>Results<br>We identified 94,180 VPH of which 41% were influenza, 33% hepatitis B and 10% herpes zoster. MH service users had more VPH admissions (aIRR 3.2, 95% CI 3.1 - 3.3). Relative risks were highest for hepatitis (aIRR 4.4, 95% CI 4.3 – 4.6), but elevated for all conditions including COVID-19 (aIRR 2.0, 95% CI 1.9 – 2.2). MH service users had a mean age nine years younger than other NSW residents at first VPH admission, with the largest age gap for vaccine-preventable pneumonias (11-13 years younger). The highest relative risk of VPH was among MH service users aged 45-65.<br>Conclusions<br>Mental health service users have increased risk of hospitalisation for many vaccine-preventable conditions. This may be due to reduced vaccination rates, more severe

Powered by Editorial Manager® and ProduXion Manager® from Aries Systems Corporation

	illness requiring hospitalisation, greater exposure to infectious conditions, or other factors. People living with mental illness should be prioritised in vaccination strategies.

Powered by Editorial Manager® and ProduXion Manager® from Aries Systems Corporation

Main Document (manuscript)

Click here to access/download;Main Document (manuscript);VPH changes accepted 23_03_07 Main - Ref

Ms. No. PSM-D-22-01657 (Revised)

Vaccine-preventable hospitalisations in adult mental health service users: a population study.

Grant Sara, Patrick Gould, Jackie Curtis, Wendy Chen, Michael Lau, Parashar Ramanuj, David Currow, Philip Burgess.

Author for correspondence: Grant Sara. Email Grant.Sara@health.nsw.gov.au

Key words: Vaccination, Epidemiology, Prevention

Funding Statement: This research received no specific grant from any funding agency, commercial or not-for-profit sectors

Ethical standards: This work does not involve human and/or animal experimentation. The study was approved by the NSW Population and Health Services Research Ethics Committee (Approval references 2017/HRE1105, 2018/UMB0304).

Word count: Abstract 239. Body 4342 (including in text citations)

Abstract

Background

Vaccine-preventable conditions cause preventable illness and may increase mortality in people living with mental illness. We examined how risks of hospitalisation for a wide range of vaccine-preventable conditions varied by age and sex among mental health service users.

Methods

Linked population data from New South Wales (NSW), Australia were used to identify vaccine-preventable hospitalisations (VPH) for 19 conditions from 2015 to 2020. Adult mental health (MH) service users (n=418,915) were compared to other NSW residents using incidence rates standardised for age, sex and socioeconomic status. Secondary analyses examined admissions for COVID-19 to Sept 2021.

Results

We identified 94,180 VPH of which 41% were influenza, 33% hepatitis B and 10% herpes zoster. MH service users had more VPH admissions (aIRR 3.2, 95% CI 3.1 - 3.3). Relative risks were highest for hepatitis (aIRR 4.4, 95% CI 4.3 – 4.6), but elevated for all conditions including COVID-19 (aIRR 2.0, 95% CI 1.9 – 2.2). MH service users had a mean age nine years younger than other NSW residents at first VPH admission, with the largest age gap for vaccine-preventable pneumonias (11-13 years younger). The highest relative risk of VPH was among MH service users aged 45-65.

Conclusions

Mental health service users have increased risk of hospitalisation for many vaccine-preventable conditions. This may be due to reduced vaccination rates, more severe illness requiring hospitalisation, greater exposure to infectious conditions, or other factors. People living with mental illness should be prioritised in vaccination strategies.

Background

People living with mental ill health experience increased rates of chronic and preventable medical conditions, avoidable hospitalisations and premature mortality (Firth *et al.*, 2019). Vaccine-preventable conditions such as influenza, pneumonia, hepatitis and COVID-19 are likely to contribute to this morbidity and mortality.

People living with mental ill health have low vaccination rates. Studies in clinical cohorts with severe mental illness have found influenza vaccination rates from 25% (Lorenz *et al.*, 2013) to as low as 7% (Young *et al.*, 2015), and vaccination rates below 10% for pneumococcal pneumonia, measles and hepatitis (Miles *et al.*, 2020). Among US veterans, those with mental health conditions are up to twenty percent less likely to receive vaccination for pneumococcal disease or influenza (Druss *et al.*, 2002). In Israel, vaccination rates against COVID-19 were 25% lower in people with non-affective psychoses than in the broader population (Goldberger *et al.*, 2022).

Rates of vaccine-preventable conditions are also increased in people living with mental health conditions. In recent meta-analyses, people with serious mental illness had up to five-fold increases in the prevalence of Hepatitis B (Ayano *et al.*, 2018, Hughes *et al.*, 2016), with higher rates in men (Ayano *et al.*, 2018) and in people with a diagnosis of schizophrenia (Lluch and Miller, 2019). Fewer studies have examined other vaccine-preventable conditions. In Taiwan's national claims database, people with a broad range of mental health conditions were 30% more likely to be diagnosed with Herpes Zoster (Yang *et al.*, 2011), and people diagnosed with bipolar disorder were more than twice as likely to be diagnosed with pneumonia or tuberculosis (Chen *et al.*, 2022). A population study from Israel found that people with a mental health condition were less likely to be diagnosed with COVID-19, but this may have reflected lower testing rates (Goldberger *et al.*, 2022).

Few studies have examined hospitalisations or deaths due to vaccine-preventable illnesses in large, representative populations of people with a mental health condition. Studies from England, Sweden and Denmark have suggested a two to three times increased risk of hospitalisation for influenza and pneumonia in mental health service users, even when controlling for confounders such as smoking status (Crump *et al.*, 2013, Davydow *et al.*, 2016, Seminog and Goldacre, 2013). In Israel, people with mental health diagnoses had more than twice the population risk of hospitalisation or death after diagnosis with COVID-19 (Goldberger *et al.*, 2022). Two Australian studies have examined sub-national (state) populations, finding a two- to four-fold increased risk of hospitalisation for vaccine-preventable conditions including influenza, pneumonia and hepatitis (Mai *et al.*, 2011, Sara *et al.*, 2021).

More evidence about vaccine-preventable hospitalisations is required to understand the scale of this issue and to identify priority areas or groups for action. Most studies have focused on hospitalisations for respiratory illness. No study has reported on a broad range of specific vaccine-preventable conditions or presented age-specific risks. Many of the studies above include data from the 1960s to the early 2000s, however we need updated data because of new vaccine approaches and new patterns of illness, including COVID 19.

The current study describes hospitalisation rates for vaccine-preventable conditions in the state of New South Wales (NSW), Australia. We examine hospitalisation rates over five years for nineteen specific vaccine-preventable conditions. We compare rates for the overall adult population to those in mental health service users, and report age- and sex-specific rates adjusting for differences in socio-economic disadvantage.

Methods

Study design

All vaccine-preventable hospitalisations (VPH) were identified over five years in the state of New South Wales (NSW), Australia using retrospective observational methods. The outcome of interest was the number of vaccine-preventable hospitalisations (episodes of hospital care) (VPH) and the number of VPH related hospital days, expressed as rates per 100,000 population per year.

Data linkage

Data from NSW public hospitals, private hospitals, public community mental health services and the NSW Register of Births Deaths and Marriages were linked by the NSW Centre for Health Record Linkage (CHeReL), using probabilistic record linkage based on individuals' names, date of birth, addresses and health service identifiers. The linkage process is designed to give a false positive linkage rate of around 5 per 1000 records. More detail on the datasets and linkage methods is provided elsewhere (Sara *et al.*, 2019)

Study setting

Vaccine-preventable hospitalisations to NSW private and government-funded (public) hospitals were examined. Around 60% of Australian hospital care is provided through state operated public hospitals (Australian Institute of Health and Welfare, 2022, December 7). Private hospitals mainly provide non-emergency care for individuals opting-in to private health insurance. Hospital diagnoses are recorded using the International Classification of Diseases, 10th Edition, Australian Modification (National Centre for Classification in Health, 2010).

Mental health service users were defined by receiving any mental health care from a NSW public or private hospital, or a state government operated (public) community mental health services. Public community mental health services provide care to around 2% of the NSW population each year (Australian Institute of Health and Welfare, 2022, December 12), focusing on emergency care and longer term care of severe or complex disorders. Primary and private mental health outpatient services see around 11% of the NSW population each year (Australian Institute of Health and Welfare, 2022, December 12), and focus mainly on higher prevalence mental health conditions: data for those services are not included in the current linkage.

Primary outcome: Vaccine-preventable hospitalisations

The main outcome was admission to any NSW public or private hospital with a primary or secondary diagnosis of a vaccine-preventable hospitalisation (VPH) during a five-year period from 1 July 2015 to 30 June 2020. We examined 19 vaccine-preventable conditions, using specifications of the Australian Commission on Quality and Safety in Health Care (Falster and Jorm, 2017) supplemented with additional codes. These were divided into three subgroups: (i) respiratory conditions (influenza, pneumococcal pneumonia, haemophilus pneumonia, pertussis, diphtheria, COVID-19); (ii) hepatitis B, and; (iii) other vaccine-preventable conditions (herpes zoster, tuberculosis, varicella, rotaviral enteritis, mumps, measles, haemophilus meningitis, tetanus, rubella, cholera, acute poliomyelitis). Hepatitis B was grouped separately because of its high prevalence and because its mode of transmission and associations were assumed to be different from those of other conditions. Hepatitis C was not included as it is not currently vaccine preventable.

Mental health cohort definition

Mental health service users were defined as NSW residents aged 18-100 years who had any mental health hospitalisation or public community mental health contact between 1 July 2013 and 30 June 2020. Mental health hospitalisations were defined as episodes of care in a public or private hospital with a primary diagnosis of a non-organic mental health condition (ICD-10 codes F10-F99) or at least one day in a designated mental health unit. Community contacts included face-to-face or telehealth contacts with NSW public community mental health services. Services to non-NSW residents, administrative contacts, case conferences and contacts by community teams with hospital inpatients were excluded. Sex and area of residence were defined at the first (index) contact in the observation period. Age was estimated at the midpoint of the study period. Mental health status was treated as time-dependent, with events (hospitalisations) and exposure time (person years) calculated from the date of each person's index mental health contact in the study period, or from the start of the study period (1 Jul 2015) if the index contact occurred during the two-year pre-study look-back period. Deaths during the study period were identified by linkage to the NSW Register of

Births, Deaths and Marriages. If death occurred during the study period, time from the date of death to the study end was excluded when calculating hospitalisation rates.

Data analysis

Data assembly and standardisation were conducted in SAS Enterprise Guide v7.15. VPH rates per 100,000 person-years were calculated for Mental Health (MH) and non-MH groups. VPH episode and day rates were calculated for (i) any VPH, (ii) three VPH groups (respiratory, hepatitis B, other), and (iii) 19 individual VPH conditions. Episodes with multiple VPH diagnoses were counted separately for condition-specific rates but treated as a single episode when calculating overall VPH rates.

For calculation of standardised rates, admission rates were first calculated separately for each study group, subgroup, vaccine preventable condition group, individual vaccine preventable condition, and stratum of age (18-24,25-34,35-44,45-54,55-64,65-75,75-84,85+), sex and quintile of socioeconomic disadvantage. Socioeconomic disadvantage was estimated from the person's area of residence, using the Australian Bureau of Statistics Index of Relative Socioeconomic Disadvantage (IRSD) (Australian Bureau of Statistics, 2006). This index is calculated for Australian geographical areas using 17 census-derived variables measuring income, government welfare support, education, home ownership, employment, household structure and English language proficiency. Denominators for rate calculations were (i) for mental health service users: stratum-specific populations from the mental health cohort and (ii) for other NSW residents: census-derived population estimates from the Australian Bureau of Statistics for the midpoint of the study period, after subtracting the relevant mental health service user population. Adjusted Incidence Rate Ratios (aIRRs) and 95% Log Normal confidence intervals were then calculated by direct standardisation for age, sex and socioeconomic status, using the SAS procedure "Proc STDRATE" (SAS Institute Inc, 2020, October 28). Standardised rates were not calculated where the number of VPH events in the MH cohort was less than 20.

A supplementary analysis was conducted to examine admission rates for COVID-19, using public hospital data from July 2020 to September 2021. The original planned analysis included all NSW public and private hospitals, however private hospital data were available only to June 2020. That covered three months of the first wave of the NSW pandemic (April to June 2020), during which hospital admissions for COVID-19 were uncommon. Public hospital data were also available to September 2021, which included the peak of the much larger NSW COVID-19 Delta wave. Rates and adjusted rate ratios were calculated using the same methods and population denominators as the main analyses.

Two subgroup analyses were conducted. First, we examined VPH rates in mental health service users with severe or persistent mental illness (SPMI), who were defined as people with (i) any diagnosis of schizophrenia, schizoaffective disorder, bipolar disorder or psychotic depression, or (ii) more than two years of contact with mental health services, as measured from the dates of their first and last mental health service contacts during the observation and pre-study lookback periods. Second, we examined VPH rates in mental health service users who had received hospital care, compared to people whose only mental health service contact was with community-based, non-admitted mental health services.

Ethics approval

The study was approved by the NSW Population and Health Service Research Ethics Committee (HREC/17/CIPHS/48. CINSW Refs 2017/HRE1105, 2019/UMB0208), and the Aboriginal Health and Medical Research Council of NSW (Ref 1564/19).

Results

We identified 500,548 people who had at least one in-scope contact with NSW mental health services in the study or look-back periods. Of these, we excluded 62,514 (12.5%) people aged under 18 at first contact, 12,987 (2.6%) with no valid age recorded, and 72 (0.01%) with sex missing or recorded as "other than male or female". We also excluded 5,706 people (1.1%) who had contact in the pre-study look-back period but died before the start of the observation period. After these exclusions 418,915 mental health service users were included in calculation of standardised rates. Nineteen percent had a diagnosis of a psychotic disorder recorded, and approximately half (51%) received community mental health care only (see Supplementary Table 1). Compared to NSW population estimates for the mid-point of the study period (Jan 2018), mental health service users were more likely to be female, aged under 45, and to live in regions in the most disadvantaged two quintiles of the NSW population (Table 1).

During the study period there were 94,180 vaccine-preventable hospitalisations (VPH) to NSW public or private hospitals (Table 2). Nearly all (89%) occurred in public hospitals. These hospitalisations included 97,910 individual vaccine-preventable condition diagnoses, because a small number of admissions included more than one such diagnosis. The most frequently diagnosed individual conditions were influenza (41% of all VPH diagnoses), hepatitis B (33%) and herpes zoster (10%). These three conditions, along with two vaccine-preventable pneumonias (haemophilus pneumoniae, streptococcus pneumoniae) made up 98% of all VPH diagnoses. The study period for the primary analysis included only the first three months of the COVID-19 pandemic; 668 COVID admissions were recorded, including 27 in mental health service users. Conditions with fewer than 20

admissions in the mental health cohort are not reported separately but have been included in group and state totals. These include mumps (109 total admissions), measles (30), haemophilus meningitis (24), tetanus (16), rubella (12), cholera (10), diphtheria (9) and acute poliomyelitis (9).

Compared to other NSW residents, mental health service users were 2.6 times more likely to experience a vaccine-preventable hospitalisation, with an aIRR of 3.2 (95% CI 2.1 – 2.2) after standardising for age, sex and socioeconomic disadvantage. Relative risk was increased for all VPH types other than COVID-19, with the highest relative risk for hepatitis B (aIRR 4.4, 95% CI 4.3 – 4.6).

A supplementary analysis was conducted for the period from July 2020 to September 2021 to examine hospitalisation rates for COVID, using data for NSW public hospitals only (Table 3). During this period there were 10,186 admissions for COVID-19 to NSW public hospital and “hospital in the home” services (1,243 in mental health service users, 8,943 in other NSW residents). Overall VPH admission rates fell during this period, dropping by 21% in the broader population and 12% in mental health service users, with substantial reductions in admission for most VPH types and almost complete absence of influenza hospitalisations. In this period, mental health users were twice as likely to have a hospital admission with a COVID-19 diagnosis (aIRR 2.0, 95% CI 1.9 – 2.2).

Patterns of vaccine-preventable hospitalisation differed by age, sex and type of vaccine-preventable condition (Figure 1 and Supplementary Table 2). In the broader NSW population VPH rates approximately doubled for each decade over age 55. In mental health service users, rates increased from a younger age and the highest relative risks occurred in mental health service users aged 35-64. Mental health service users aged 35-54 had very high relative risks of admission for hepatitis B. In mental health service users aged 75 and above, relative risks converged towards population rates.

Earlier age-related increases in risk of VPH admission were also reflected in younger average age at admission for mental health service users. On average, mental health service users were 9 years younger than other NSW residents when admitted with a VPH diagnosis (Figure 2). This age gap was largest for respiratory conditions, particularly for pneumonia due to haemophilus (13 years) or streptococcus (11 years).

Examining the secondary outcome of hospital bed days, mental health service users had a slightly longer average length of hospital stay for vaccine-preventable conditions (MH service users 9.7 days, other NSW residents 8.1 days). Because of the combination of a longer length of stay and a higher hospitalisation rate, mental health service users experienced 3.8 times more hospital bed days per capita than other NSW residents (95% CI 3.77 – 3.82) after adjusting for age, sex and socioeconomic status (Supplementary Table 3).

In subgroup analysis, people with severe or persistent mental illness (SPMI) made up 33% of people and 40% of person-years in the mental health cohort (Supplementary Table 4). Standardised VPH rates were highest in the SPMI group (aIRR 4.0, 95% CI 3.9-4.1), but also significantly elevated in other mental health service users (aIRR 2.8, 95% CI 2.8-2.9) compared to other NSW residents. The highest relative risk in the SPMI group was for Hepatitis B (aIRR 6.1, 96% CI 5.8 - 6.3). Both groups of mental health service users had significantly increased risk of hospitalisation for all disorders examined. The SPMI subgroup had significantly higher standardised VPH rates than the other mental health subgroup overall, for hepatitis and respiratory conditions. However, the other mental health subgroup had higher standardised VPH rates than the SPMI subgroup for other vaccine-preventable conditions, including herpes zoster, tuberculosis and varicella. A similar pattern was seen in subgroup analysis based on the type of mental health care received (Supplementary Table 5). Compared to non-mental health-service users, the relative risk of any VPH was slightly higher in people with any mental health hospital care (aIRR 3.6, 95% CI3.5-3.7) than in people receiving community mental health care only (aIRR 3.1, 95% CI 3.0 – 3.2). Compared to people with community mental health care only, people with admitted mental health care had a slightly higher rate of VPH for hepatitis and respiratory conditions but slightly lower rate for other vaccine-preventable conditions.

Discussion

We compared hospitalisation rates for 19 individual vaccine-preventable conditions in 418,915 adult mental health service users to a broader population of 6.2 million adults over a five-year period. Each year in NSW there were nearly 20,000 VPH, occupying more than 120,000 bed days. After adjusting for differences in age, sex and socioeconomic disadvantage, mental health service users were 3.2 times more likely than other NSW residents to be admitted to hospital for a vaccine-preventable condition and had 3.8 times more days in hospital per capita for those conditions. The highest relative risk in mental health service users was for hepatitis B, but VPH rates were increased across the spectrum of vaccine-preventable conditions, including COVID-19. The rate of VPH was slightly higher in the subgroup of mental health service users with severe and persistent mental illness, but other mental health service users also had significantly increased rates.

Previous studies have found that people with prior mental health service contact were two to three times more likely to be admitted for influenza and pneumonia (Crump *et al.*, 2013, Mai *et al.*, 2011, Seminog and Goldacre, 2013). We found a 2.8 fold increase in admission rate for respiratory conditions, similar to findings of Crump (Crump *et al.*, 2013) and Davydow (Davydow *et al.*, 2016). We also found similar increases for other respiratory conditions including pertussis, as well as for

herpes zoster and varicella. Our main analysis covered only the first few months of the COVID-19 pandemic in NSW and did not show increased hospitalisation for COVID-19. However secondary analysis of a longer period of public hospital data found a two-fold risk of COVID admission in mental health service users, consistent with recent findings from Israel (Goldberger *et al.*, 2022). These findings of increased hospitalisation rates across a broad spectrum of conditions suggest that causes may also be broad, and that prevention strategies may need to consider diverse conditions and risk groups.

We found that the normal age-related risk curve for vaccine-preventable hospitalisations appears to be shifted to the left in mental health service users. Service users were, on average, almost a decade younger when admitted for vaccine-preventable conditions. Mental health service users in their 40s and 50s had particularly elevated risk of admission for vaccine-preventable respiratory conditions. For effective prevention, public health strategies may need to consider the earlier onset of chronic medical illness in this group (Firth *et al.*, 2019). In Australia, as in many countries, mental health conditions are not currently considered amongst criteria for subsidised access to vaccination at a younger age than the general public for conditions such as influenza, pneumococcal pneumonia or herpes zoster (Australian Technical Advisory Group on Immunisation, 2023, January 25). Hepatitis B vaccines are only subsidised for those under 20 years old or for refugee backgrounds. Influenza vaccines are subsidised for those over 65 years old, Aboriginal and Torres Strait Islander people (Australian Technical Advisory Group on Immunisation, 2023, January 25), and people with selected medical conditions. Severe mental illness is not included in the list of medical conditions allowing subsidised vaccine access (Australian Technical Advisory Group on Immunisation, 2023, January 25). This creates a significant barrier to service providers and individuals in increasing adult vaccination for this population. By contrast, in New Zealand free access to influenza vaccines has been expanded to include people living with a severe mental illness or accessing a mental health service (Manatu Hauora New Zealand Ministry of Health, 2023, March 6).

Our findings suggest that vaccine-preventable illnesses cause significant harm in people living with mental health conditions, and that increased hospitalisation risks are not simply explained by differences in age, sex or socioeconomic status. More study is needed to demonstrate the mechanisms of increased vaccine-preventable hospitalisations in this group. We do not currently have data on vaccination status to explore these interactions in our study population.

We speculate that increased hospitalisations arise through interactions between increased exposure, reduced vaccination, more severe illness, greater medical comorbidity and other risk factors. Therefore improving vaccination rates is likely to be an important but not sufficient strategy

for reducing vaccine-preventable harms in mental health service users. We need strategies to reduce policy barriers, increase access and improve vaccination coverage. We also need to understand and address the many other risk factors likely to also contribute to the development of more severe vaccine-preventable conditions in this group.

Increased rates of Hepatitis B in people with severe mental illness may reflect greater exposure through injecting drug use (Hughes *et al.*, 2016, Lluch and Miller, 2019), and for many people exposure to hepatitis B may have occurred prior to the development of mental illness. Additionally, this group may also have greater exposure to respiratory infections due to housing conditions or exposures in health-care settings. Low vaccination rates are likely to contribute to high rates of admission for respiratory conditions (Druss *et al.*, 2002, Miles *et al.*, 2020), but no studies have reported both vaccination rates and harms in the same individuals. People living with mental health conditions may have other risk factors and comorbidities causing more severe illness once exposed to infection (Firth *et al.*, 2019). For example, smoking rates are increased in people living with mental health conditions, and smoking is a risk factor for more severe illness and ICU admission following respiratory infections, including COVID (Vardavas and Nikitara, 2020). However, some studies have found that increased hospitalisation risk for respiratory illness persists after adjusting for smoking status and comorbid medical or substance use conditions (Crump *et al.*, 2013, Davydow *et al.*, 2016, Mai *et al.*, 2011), suggesting that other factors also contribute. The longer length of stay for VPH admissions in our study may reflect more severe illness, social or other barriers to discharge, or a combination of these factors. There has also been speculation that psychological distress or depressive symptoms may reduce the immune response to vaccination, increasing the risk of illness even when individuals are vaccinated (Abdeljaber *et al.*, 1994, Ford *et al.*, 2019, Glaser *et al.*, 2003, Segerstrom *et al.*, 2012, Wang *et al.*, 2016).

Strengths and limitations

This study identifies all vaccine-preventable hospital admissions for a population of more than 6 million adults and examines a population-wide cohort of people with mental health conditions. This allows a whole population view of a wide range of specific vaccine-preventable conditions. However, there are several limitations to the datasets used in this study.

First, we do not currently have linked data on vaccination status, and so cannot examine the relationship between vaccination status and increased hospitalisation rates.

Second, our dataset does not include community mental health service contacts with primary care or private mental health providers. Our cohort was defined using hospital and public community mental health data and is likely to represent people with more severe or longstanding illness sufficient to require hospital admission or be treated by public community services. They may not be representative of broader groups receiving care only in primary care or private settings for conditions such as depression or anxiety.

Third, our dataset does not include medication prescription data, and we were therefore unable to examine whether psychotropic medications were associated with vaccine preventable hospitalizations. A recent meta-analysis found that exposure to a wide range of psychotropics was associated with increased COVID-related mortality (Vai *et al.*, 2021). However, this may reflect risk associated with the conditions for which those medications were prescribed, rather than independent effects of the medications. Studies controlling for clinical and demographic confounders have reported complex interactions between medications and COVID susceptibility or severity. In people with severe mental illness mood stabilisers were associated with increased risk of COVID infection, while Clozapine and Paliperidone were associated with reduced risk. In a large emergency and acute care cohort, (Oskotsky *et al.*, 2021) prior prescription of a selective serotonin reuptake inhibitor was associated with reduced risk of COVID mortality after adjusting for demographic, clinical, co-morbidity and treatment setting variables. More study is needed to understand possible harmful or protective effects of psychotropic medications on vaccine-preventable illnesses (Nemani *et al.*, 2021).

Fourth, community mental health records in our large administrative dataset often lack diagnostic information. We therefore combined diagnosis and duration of service contact to define a subgroup with severe or persistent mental illness. While this is a common approach in operational definitions of serious mental illness (Gonzales *et al.*, 2022), it causes imprecision in our subgroup analyses because many people not meeting the operational definition of severe and persistent mental illness may still have had significant risk or impairment.

Finally, our current dataset does not cover the significant COVID-19 Omicron variant wave, which in NSW peaked in the last quarter of 2021 and early 2022. Within the NSW health system, models of care and patterns of hospitalisation for COVID continue to evolve. We plan to revise our current estimates for COVID as further data become available in future linkage cycles

Conclusions

COVID-19 reminds us that vaccine programs are essential in reducing the impact of preventable infections on individuals and health systems. There have been recent calls to ensure that people

living with mental illness are prioritised in the development of COVID vaccination strategies (De Hert *et al.*, 2021) and have equitable access to all vaccination programmes (Equally Well Alliance, 2021, October 1).

We need ongoing study of the mechanisms and impacts of vaccine-preventable harms in people living with mental illness, to identify priority conditions and groups, and to evaluate effective models for intervention.

References

Abdeljaber, M. H., Nair, M. P. N., Schork, M. A. & Schwartz, S. A. (1994). Depressed natural killer cell activity in schizophrenic patients. *Immunological Investigations*, 23, 259-268. doi: https://doi.org/10.3109/08820139409066822

Australian Bureau of Statistics. (2006). *An introduction to socio-economic indexes for areas (SEIFA).* Commonwealth of Australia: Canberra.

Australian Institute of Health and Welfare. (2022, December 7). Hospital activity. Retrieved from https://www.aihw.gov.au/reports/hospitals/australias-hospitals-at-a-glance/contents/hospital-activity

Australian Institute of Health and Welfare. (2022, December 12). Mental health services in Australia: national healthcare agreement indicators. Retrieved from https://www.aihw.gov.au/reports/mental-health-services/mental-health-services-in-australia/report-contents/mental-health-indicators/national-healthcare-agreement-indicators

Australian Technical Advisory Group on Immunisation. (2023, January 25). Australian immunisation handbook. Retrieved from https://immunisationhandbook.health.gov.au/

Ayano, G., Tulu, M., Haile, K., Assefa, D., Habtamu, Y., Araya, G. & Yohannis, Z. (2018). A systematic review and meta-analysis of gender difference in epidemiology of HIV, hepatitis B, and hepatitis C infections in people with severe mental illness. *Annals of General Psychiatry*, 17. doi: https://doi.org/10.1186/s12991-018-0186-2

Chen, P. H., Tsai, S. Y., Pan, C. H., Chen, Y. L., Su, S. S., Chen, C. C. & Kuo, C. J. (2022). Prevalence and 5-year trend of incidence for medical illnesses after the diagnosis of bipolar disorder: A nationwide cohort study. *Australian & New Zealand Journal of Psychiatry*, 56, 1164-1176. doi: https://doi.org/10.1177/00048674211046891

Crump, C., Sundquist, K., Winkleby, M. A. & Sundquist, J. (2013). Comorbidities and mortality in bipolar disorder: A Swedish national cohort study. *JAMA Psychiatry*, 70, 931-939. doi: https://doi.org/10.1001/jamapsychiatry.2013.1394

Davydow, D. S., Ribe, A. R., Pedersen, H. S., Fenger-Gron, M., Cerimele, J. M., Vedsted, P. & Vestergaard, M. (2016). Serious mental illness and risk for hospitalizations and rehospitalizations for ambulatory care-sensitive conditions in Denmark: A nationwide population-based cohort study. *Medical Care*, 54, 90-7. doi: https://doi.org/10.1097/MLR.0000000000000448

De Hert, M., Mazereel, V., Detraux, J. & Van Assche, K. (2021). Prioritizing COVID-19 vaccination for people with severe mental illness. *World Psychiatry*, 20, 54-55. doi: https://doi.org/10.1002/wps.20826

Druss, B. G., Rosenheck, R. A., Desai, M. M. & Perlin, J. B. (2002). Quality of preventive medical care for patients with mental disorders. *Medical Care*, 40, 129-36. doi: https://doi.org/10.1097/00005650-200202000-00007

Equally Well Alliance. (2021, October 1). Fair and equitable access to vaccination for people living with mental illness and substance use disorders: a global call to action. Retrieved from https://www.equallywell.org.au/declaration/

Falster, M. & Jorm, L. (2017). *A guide to the potentially preventible hospitalisations indicator in Australia.* University of New South Wales in consultation with Australian Commission on Safety and Quality in Health Care and Australian Institute of Health and Welfare,: Sydney.

Firth, J., Siddiqi, N., Koyanagi, A., Siskind, D., Rosenbaum, S., Galletly, C., ... Stubbs, B. (2019). The Lancet Psychiatry Commission: a blueprint for protecting physical health in people with mental illness. *The Lancet Psychiatry*, 6, 675-712. doi: https://doi.org/10.1016/S2215-0366(19)30132-4

Ford, B. N., Yolken, R. H., Dickerson, F. B., Teague, T. K., Irwin, M. R., Paulus, M. P. & Savitz, J. (2019). Reduced immunity to measles in adults with major depressive disorder. *Psychological Medicine*, 49, 243-249. doi: https://doi.org/10.1017/S0033291718000661

Glaser, R., Robles, T. F., Sheridan, J., Malarkey, W. B. & Kiecolt-Glaser, J. K. (2003). Mild depressive symptoms are associated with amplified and prolonged inflammatory responses after influenza virus vaccination in older adults. *Archives of General Psychiatry*, 60, 1009-14. doi: https://doi.org/10.1001/archpsyc.60.10.1009

Goldberger, N., Bergman-Levy, T., Haklai, Z., Yoffe, R., Davidson, M., Susser, E., ... Weiser, M. (2022). COVID-19 and severe mental illness in Israel: testing, infection, hospitalization, mortality and vaccination rates in a countrywide study. *Molecular Psychiatry*, 27, 3107-3114. doi: https://doi.org/10.1038/s41380-022-01562-2

Gonzales, L., Kois, L. E., Chen, C., Lopez-Aybar, L., McCullough, B. & McLaughlin, K. J. (2022). Reliability of the term "serious mental illness": A systematic review. *Psychiatric Services*, 73, 1255-1262. doi: https://doi.org/10.1176/appi.ps.202100661

Hughes, E., Bassi, S., Gilbody, S., Bland, M. & Martin, F. (2016). Prevalence of HIV, hepatitis B, and hepatitis C in people with severe mental illness: a systematic review and meta-analysis. *The Lancet Psychiatry*, 3, 40-48. doi: https://doi.org/10.1016/s2215-0366(15)00357-0

Lluch, E. & Miller, B. J. (2019). Rates of hepatitis B and C in patients with schizophrenia: A meta-analysis. *General Hospital Psychiatry*, 61, 41-46. doi: https://doi.org/10.1016/j.genhosppsych.2019.10.007

Lorenz, R. A., Norris, M. M., Norton, L. C. & Westrick, S. C. (2013). Factors associated with influenza vaccination decisions among patients with mental illness. *International Journal of Psychiatry in Medicine*, 46, 1-13. doi: https://doi.org/10.2190/PM.46.1.a

Mai, Q., Holman, C. D., Sanfilippo, F. M. & Emery, J. D. (2011). The impact of mental illness on potentially preventable hospitalisations: a population-based cohort study. *BMC Psychiatry*, 11, 163. doi: https://doi.org/10.1186/1471-244X-11-163

Manatu Hauora New Zealand Ministry of Health. (2023, March 6). 2023 free flu vaccine criteria. Retrieved from https://www.health.govt.nz/your-health/conditions-and-treatments/diseases-and-illnesses/influenza/flu-influenza-vaccines

Miles, L. W., Williams, N., Luthy, K. E. & Eden, L. (2020). Adult vaccination rates in the mentally ill population: An outpatient improvement project. *Journal of the American Psychiatric Nurses Association*, 26, 172-180. doi: https://doi.org/10.1177/1078390319831763

National Centre for Classification in Health. (2010). *The international statistical classification of diseases and related health problems, tenth revision, Australian modification (7th edn).*

National Centre for Classification in Health, Faculty of Health Sciences, The University of Sydney: Sydney.

Nemani, K., Li, C., Olfson, M., Blessing, E. M., Razavian, N., Chen, J., ... Goff, D. C. (2021). Association of psychiatric disorders with mortality among patients with COVID-19. *JAMA Psychiatry*, 78, 380-386. doi: https://doi.org/10.1001/jamapsychiatry.2020.4442

Oskotsky, T., Maric, I., Tang, A., Oskotsky, B., Wong, R. J., Aghaeepour, N., ... Stevenson, D. K. (2021). Mortality risk among patients With COVID-19 prescribed selective serotonin reuptake inhibitor antidepressants. *JAMA Network Open*, 4, e2133090. doi: https://doi.org/10.1001/jamanetworkopen.2021.33090

Sara, G., Arumuganathan, M., Chen, W., Wu, F., Currow, D., Large, M., ... Burgess, P. M. (2019). Cohort profile: Mental Health Living Longer: a population-wide data linkage to understand and reduce premature mortality in mental health service users in New South Wales, Australia. *BMJ Open*, 9, e033588. doi: https://doi.org/10.1136/bmjopen-2019-033588

Sara, G., Chen, W., Large, M., Ramanuj, P., Curtis, J., McMillan, F., ... Burgess, P. (2021). Potentially preventable hospitalisations for physical health conditions in community mental health service users: a population-wide linkage study. *Epidemiology and Psychiatric Sciences*, 30, e22. doi: https://doi.org/10.1017/S204579602100007X

SAS Institute Inc. (2020, October 28). The STDRATE procedure. Retrieved from https://documentation.sas.com/doc/en/statug/15.2/statug_stdrate_toc.htm

Segerstrom, S. C., Hardy, J. K., Evans, D. R. & Greenberg, R. N. (2012). Vulnerability, distress, and immune response to vaccination in older adults. *Brain and Behavioural Immunology*, 26, 747-53. doi: https://doi.org/10.1016/j.bbi.2011.10.009

Seminog, O. O. & Goldacre, M. J. (2013). Risk of pneumonia and pneumococcal disease in people with severe mental illness: English record linkage studies. *Thorax*, 68, 171-6. doi: https://doi.org/10.1136/thoraxjnl-2012-202480

Vai, B., Mazza, M. G., Delli Colli, C., Foiselle, M., Allen, B., Benedetti, F., ... De Picker, L. J. (2021). Mental disorders and risk of COVID-19-related mortality, hospitalisation, and intensive care unit admission: a systematic review and meta-analysis. *The Lancet Psychiatry*, 8, 797-812. doi: https://doi.org/10.1016/S2215-0366(21)00232-7

Vardavas, C. I. & Nikitara, K. (2020). COVID-19 and smoking: A systematic review of the evidence. *Tobacco Induced Diseases*, 18, 20. doi: https://doi.org/10.18332/tid/119324

Wang, Y., Yu, L., Zhou, H., Zhou, Z., Zhu, H., Li, Y., ... Dong, C. (2016). Serologic and molecular characteristics of hepatitis B virus infection in vaccinated schizophrenia patients in China. *Journal of Infection in Developing Countries*, 10, 427-31. doi: https://doi.org/10.3855/jidc.7377

Yang, Y. W., Chen, Y. H. & Lin, H. W. (2011). Risk of herpes zoster among patients with psychiatric diseases: a population-based study. *Journal of the European Academy of Dermatology & Venereology*, 25, 447-53. doi: https://doi.org/10.1111/j.1468-3083.2010.03811.x

Young, S., Dosani, N., Whisler, A. & Hwang, S. (2015). Influenza vaccination rates among homeless adults with mental illness in Toronto. *Journal of Primary Care & Community Health*, 6, 211-4. doi: https://doi.org/10.1177/2150131914558881

Figure 1 Click here to access/download;Figure(s);Figure 1 22_10_16.docx

Figure 1: Vaccine-preventable Hospitalisations (VPH) in NSW adults aged 18-100, by age group, gender and type of vaccine-preventable condition

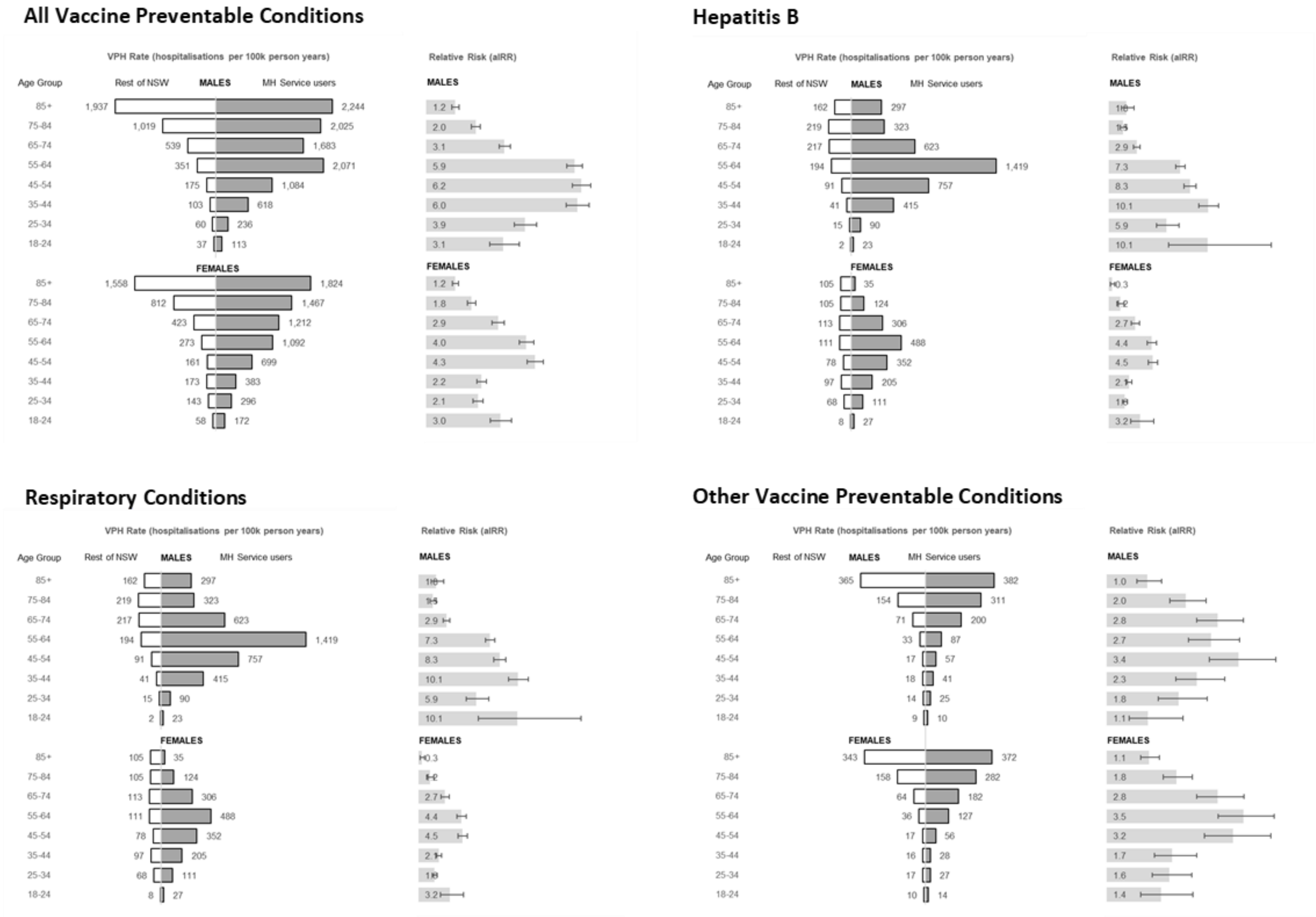

Figure 2

Click here to access/download;Figure(s);Figure 2 22_10_16.docx

Figure 2: Average age at Vaccine-preventable Hospitalisation (VPH), comparing mental health (MH) service users to other NSW residents.

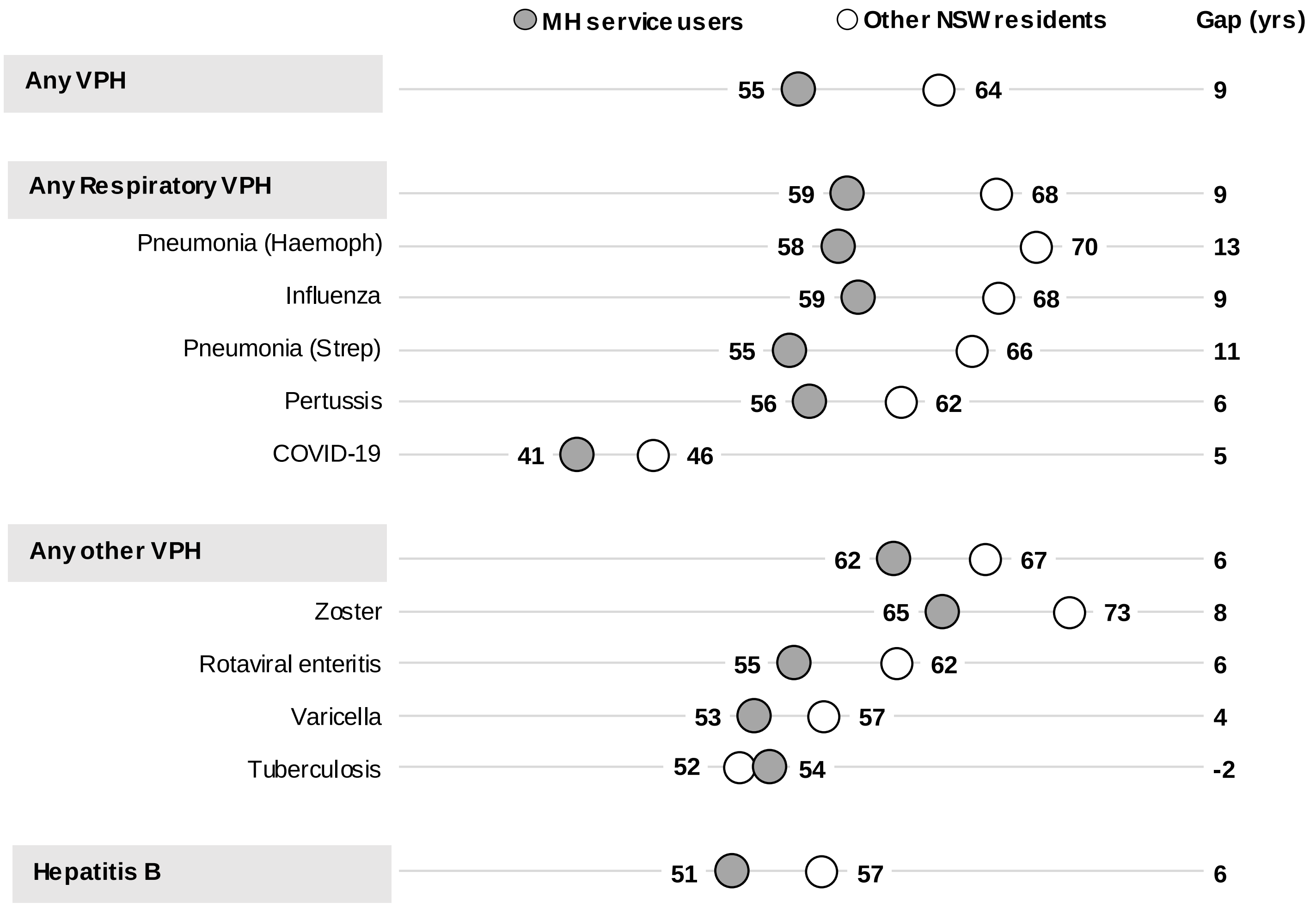

Table 1 Cohort description

	MH service users n (% of group)	NSW Population aged 18+ n (% of group)
**Total**	418,915 (100%)	6,162,602 (100%)
**Sex**		
Female	217,843 (52%)	3,137,557 (51%)
Male	201,072 (48%)	3,025,046 (49%)
**Age group**		
18-24	70,920 (17%)	733,781 (12%)
25-34	87,513 (21%)	1,186,254 (19%)
35-44	80,886 (19%)	1,050,450 (17%)
45-54	66,529 (16%)	1,001,947 (16%)
55-64	46,695 (11%)	923,220 (15%)
65-74	30,765 (7%)	708,857 (12%)
75-84	21,244 (5%)	387,496 (6%)
85+	14,363 (3%)	170,598 (3%)
**Disadvantage quintile**		
Q1 Most	70,165 (17%)	1,124,329 (18%)
Q2	116,543 (28%)	1,078,653 (18%)
Q3	76,946 (18%)	1,305,848 (21%)
Q4	68,601 (16%)	1,240,146 (20%)
Q5 Least	71,433 (17%)	1,413,626 (23%)
Unknown	15,227 (4%)	-

Notes: Mental health service users aged 18-100, and NSW adult population, July 2015 to June 2020. Estimated NSW adult population at study midpoint (Jan 2018). Disadvantage quintiles based on person's address of residence, using Index of Relative Socioeconomic Disadvantage (IRSD).

TABLE 2: Vaccine-preventable hospitalisations (VPH) of adults (aged 18-100) to NSW public and private hospitals, July 2015 to June 2020, comparing people receiving any hospital or community mental health care to the rest of the NSW population. Adjusted incidence rate ratios (aIRR) after standardisation by (1) age and sex, (2) age, sex and socioeconomic disadvantage.

	VPH episodes (n)		Crude rate (per 100k person years)			Adjusted incidence rate ratios (aIRR), (95% CI)	
	MH care	No MH care	MH care	No MH care	IRR	1. Age, Sex.	2. Age, Sex, Disadvantage
**Any Vaccine-preventable**	**10,679**	**83,501**	**726.7**	**284.6**	**2.6**	**3.1 (3.0 - 3.1)**	**3.2 (3.1 - 3.3)**
**Hepatitis B**	**4,901**	**26,371**	**333.5**	**89.9**	**3.7**	**4.2 (4.1 - 4.4)**	**4.4 (4.3 - 4.6)**
**Respiratory infections (1)**	**4,918**	**45,820**	**334.7**	**156.1**	**2.1**	**2.7 (2.6 - 2.7)**	**2.8 (2.7 - 2.9)**
Influenza	3,609	35,381	245.6	120.6	2.0	2.5 (2.5 - 2.6)	2.7 (2.6 - 2.8)
Haemophilus pneumonia	505	4,618	34.4	15.7	2.2	2.7 (2.5 - 3.0)	2.8 (2.6 - 3.1)
Streptococcal pneumonia	901	6,220	61.3	21.2	2.9	3.5 (3.2 - 3.7)	3.6 (3.3 - 3.9)
COVID	27	641	1.8	2.2	0.8	1.0 (0.6 - 1.4)	0.9 (0.6 - 1.3)
Pertussis (whooping cough)	55	449	3.7	1.5	2.4	3.0 (2.2 - 4.0)	3.1 (2.3 - 4.2)
**Other (2)**	**1,029**	**12,535**	**70.0**	**42.7**	**1.6**	**2.1 (2.0 - 2.2)**	**2.2 (2.0 - 2.3)**
Herpes Zoster	761	9,106	51.8	31.0	1.7	2.2 (2.0 - 2.4)	2.3 (2.1 - 2.5)
Tuberculosis	162	2,301	11.0	7.8	1.4	1.6 (1.4 - 1.9)	1.8 (1.5 - 2.1)
Varicella (Chicken Pox)	101	1,108	6.9	3.8	1.8	2.2 (1.8 - 2.7)	2.1 (1.7 - 2.7)
Rotaviral enteritis	39	401	2.7	1.4	1.9	2.4 (1.7 - 3.3)	2.5 (1.7 - 3.5)

Notes: VPH: Vaccine-preventable Hospitalisation. MH: mental health. Disadvantage measured using index of relative socioeconomic disadvantage (IRSD) of address of residence. (1) Respiratory group total includes 9 hospitalisations for Diphtheria. (2) Other group total includes 22 hospitalisations for Mumps, Measles, Haemophilus meningitis, Tetanus, Rubella, Cholera, Acute poliomyelitis. The sum of individual conditions may exceed group totals because some admissions have more than one VPH diagnosis recorded.

Table 3: VPH admission rates, NSW public hospitals before and during COVID-19, by VPH condition and MH service user group

	VPH episodes (n)		Rate (per 100k person years)			Adjusted Rate Ratio
PPH condition	MH service users	Other NSW residents	MH service users	Other NSW residents	IRR	aIRR (95% CI)
**July 2015 to June 2020**						
Total Vaccine-preventable	10,263	73,370	698.4	250.0	2.8	3.3 (3.3 - 3.4)
Hepatitis B	4,742	20,807	322.7	70.9	4.6	5.2 (5.0 - 5.3)
Respiratory infections	4,731	42,551	322.0	145.0	2.2	2.7 (2.7 - 2.8)
Influenza	3,456	32,742	235.2	111.6	2.1	2.6 (2.5 - 2.7)
Haemophilus pneumonia	487	4,280	33.1	14.6	2.3	2.8 (2.6 - 3.1)
Streptococcus pneumoniae	890	5,922	60.6	20.2	3.0	3.6 (3.3 - 3.9)
COVID	26	628	2.2	2.1	1.0	1.1 (0.8 - 1.7)
Other	957	11,195	65.1	38.2	1.7	2.2 (2.0 - 2.3)
Herpes Zoster	709	7,927	48.3	27.0	1.8	2.4 (2.2 - 2.6)
Tuberculosis	153	2,228	10.4	7.6	1.4	1.6 (1.3 - 1.9)
Varicella (Chicken Pox)	98	1,039	6.7	3.5	1.9	2.3 (1.8 - 2.8)
**July 2020 to September 2021 (includes COVID Delta wave)**						
Total Vaccine-preventable	2,662	16,556	550.7	221.2	2.5	2.7 (2.6 - 2.8)
Hepatitis B	988	4,495	204.4	60.1	3.4	3.9 (3.7 - 4.2)
Respiratory infections	1,466	10,030	303.3	134.0	2.3	2.2 (2.1 - 2.4)
Influenza	23	150	4.8	2.0	2.4	3.2 (2.0 - 5.1)
Haemophilus pneumonia	68	397	14.1	5.3	2.7	3.4 (2.6 - 4.5)
Streptococcus pneumoniae	155	642	32.1	8.6	3.7	4.3 (3.6 - 5.1)
COVID	1,243	8,943	257.1	119.5	2.2	2.0 (1.9 - 2.2)
Other	265	2,365	54.8	31.6	1.7	2.4 (2.1 - 2.7)
Herpes Zoster	218	1,786	45.1	23.9	1.9	2.7 (2.4 - 3.2)
Tuberculosis	30	432	6.2	5.8	1.1	1.1 (0.8 - 1.7)
Varicella (Chicken Pox)	50	282	10.3	3.8	2.7	3.4 (2.5 - 4.7)

Ms. No. PSM-D-22-01657 (Revised)

Vaccine-preventable hospitalisations in adult mental health service users: a population study.

Reviewer comments and responses

REVIEWER 1

Thanks for having the privilege to review the article. This study employed a large-scale representative sample in the state of South Wales, Australia to investigate the risk of vaccine-preventable hospitalisations (VPH) in adult mental service users relative to the other NSW residents based on a population study. The key findings of this study include (1) mental health service users had more VPH admissions. Relative risks were highest for hepatitis, but elevated for all conditions including COVID-19. (2) mental health service users had a mean age nine years younger than other NSW residents at first VPH admission, with the largest age gap for vaccine-preventable pneumonias (11-13 years younger).

1. The evidence-based researches showed the use of antipsychotics on the significant higher risk of pneumonia. Is the information available in the database? Could the use of psychotropic medications confound the risk estimation of vaccine-preventable hospitalisations between the two groups in the present study?

    RESPONSE: This is an important point. We do not have access to medication information in the current dataset. We have added a paragraph to the limitations section (p11) to summarise evidence about possible harmful or protective effects of psychotropics and acknowledge this gap in our study.

2. In this study, mental health service users were defined as NSW residents aged 18-100 years who had any mental health hospitalisation or public community mental health contact between 1 July 2013 and 30 June 2020. What are their psychiatric diagnosis (the distribution)?

    RESPONSE: Apologies for this omission. A supplementary table has been added (supplementary Table 1, p22) summarizing diagnoses and care for the mental health cohort.

3. Were the subjects stratified to psychiatric outpatients or inpatients? Maybe the psychiatric inpatients have higher risk for VPH.

    RESPONSE Approximately half of the mental health cohort received community mental health care only. We have added a subgroup analysis of these two groups (Supplementary Table 5) and updated the methods (p6) and results (p9) to include this.

    Overall , this subgroup analysis for admitted vs community care showed a similar pattern to subgroup analysis for serious or persistent mental illness (SPMI) vs other mental health service users. People with SPMI and people with any admitted mental health care had slightly greater risk of any VPH and of the most frequent VPH types (respiratory conditions and hepatitis), but risks were substantially increased in both groups compared to the broader population.

4. In statistical analysis, adjusted incidence rate ratios (aIRRs) were calculated after direct standardisation for age, sex and socioeconomic status compared to the NSW population. Could the authers more detailed information how to "adjusted" incidence rate ratio? What kind of statistical analysis was used?

RESPONSE The methods section has been expanded to provide more information on the direct standardisation method (page 6 para 2). A reference has also been added for the specific SAS procedure (Proc STDRATE) used.

5. This study reported that the relative risks were highest for hepatitis. Did hepatitis including hepatitis B and C, or others? How to explain? The relevant discussion was suggested to be added.

RESPONSE The study only included Hepatitis B. Hepatitis C was not included because it was not considered a vaccine-preventable condition. Hepatitis A was not included because of the extremely low rate of hospital admissions for this condition in NSW adults. A clarifying sentence has been added to Primary Outcomes (p5)

6. Besides, how many (proportion) mental service users had received hepatitis B vaccines? How about the other NSW residents?

RESPONSE Unfortunately, the study does not currently have access to vaccination data. This is an important limitation, which we are working to address for future data linkages in our ongoing study. We have tried to make this limitation clearer by including it in the relevant section of the discussion (p10, final para) and emphasizing it as the first part of our Limitations section.

7. This study reported that the most frequently diagnosed individual conditions were influenza (41% of all VPH diagnoses), hepatitis B (33%) and herpes zoster (10%) in mental health service users. How about the influenza and herpes zoster vaccination rates in mental health service users and the other NSW residents?

RESPONSE Unfortunately, the study does not currently have access to vaccination data (see point 6 above)

REVIEWER 2

This is an excellent and timely report on rates of vaccine preventable hospitalisations in people with mental disorders, using New South Wales hospital record linkages. The paper is well written and analysed, asks an important question, the data look good and the analyses and interpretation are appropriate. My comments are minor.

1. You reference a paper giving details of record linkages, but perhaps give the linkage rates.

RESPONSE: Additional details on the variable used in linkage and the linkage false positive rate has been added to the methods section (Data Linkage section, page 4)

2. It would be helpful to give the context of vaccine policies in NSW. Not all the diseases for which there are vaccines are part of a general population vaccine policy (e.g. influenza has age limits etc). Can you provide some supplementary data on this? The point being that there may be a range of different reasons why VPH are more common in the mental illness group - some which are addressable by policy and others which may be a reflection of vaccine hesitancy etc on the part of the individual.

RESPONSE: Thankyou for the chance to strengthen the discussion on this important point. More information has been provided about age-specific vaccine policies in the Discussion, Page 10.

3. Of course it is possible that exposure to infectious illnesses like Hepatitis B occur before a mental disorder is manifest and may be a reflection of vulnerability to mental disorder (e.g. drug use, risk taking) rather than the disorder itself. The extent of any vaccine programme which seeks to reduce VPH in people with mental disorders may be affected by these considerations.

RESPONSE: We agree, and have added this caution to the discussion on this issue (p11)

5. I would suggest a short paragraph in the discussion to address the issues raised in points 2 and 3 along the lines that improved vaccine coverage policy may not be totally effective, though are clearly strongly supported by the data.

RESPONSE We have restructured the discussion to emphasise this point, adding a short paragraph along the lines suggested (p11).

Ms. No. PSM-D-22-01657 (Revised)

Vaccine-preventable hospitalisations in adult mental health service users: a population study.

Grant Sara, Patrick Gould, Jackie Curtis, Wendy Chen, Michael Lau, Parashar Ramanuj, David Currow, Philip Burgess.

Author for correspondence: Grant Sara. Email Grant.Sara@health.nsw.gov.au

Key words: Vaccination, Epidemiology, Prevention

Funding Statement: This research received no specific grant from any funding agency, commercial or not-for-profit sectors

Ethical standards: This work does not involve human and/or animal experimentation. The study was approved by the NSW Population and Health Services Research Ethics Committee (Approval references 2017/HRE1105, 2018/UMB0304).

Word count: Abstract 246. Body 3655 (including in text citations)

Abstract

Background

Vaccine-preventable conditions cause preventable illness and may increase mortality in people living with mental illness. We examined how risks of hospitalisation for a wide range of vaccine-preventable conditions varied by age and sex among mental health service users.

Methods

Linked population data from New South Wales (NSW), Australia were used to identify vaccine-preventable hospitalisations (VPH) for 19 conditions from 2015 to 2020. Adult mental health (MH) service users (n=418,915) were compared to other NSW residents using incidence rates standardised for age, sex and socioeconomic status. Secondary analyses examined admissions for COVID-19 to Sept 2021.

Results

We identified 94,180 VPH of which 41% were influenza, 33% hepatitis B and 10% herpes zoster. MH service users had more VPH admissions (aIRR 3.2, 95% CI 3.1 - 3.3). Relative risks were highest for hepatitis (aIRR 4.4, 95% CI 4.3 – 4.6), but elevated for all conditions including COVID-19 (aIRR 2.0, 95% CI 1.9 – 2.2). MH service users had a mean age nine years younger than other NSW residents at first VPH admission, with the largest age gap for vaccine-preventable pneumonias (11-13 years younger). The highest relative risk of VPH was among MH service users aged 45-65.

Conclusions

Mental health service users have increased risk of hospitalisation for many vaccine-preventable conditions. This may be due to reduced vaccination rates, more severe illness requiring hospitalisation, greater exposure to infectious conditions, or other factors. People living with mental illness should be prioritised in vaccination strategies.

Background

People living with mental ill health experience increased rates of chronic and preventable medical conditions, avoidable hospitalisations and premature mortality (Firth *et al.*, 2019). Vaccine-preventable conditions such as influenza, pneumonia, hepatitis and COVID-19 are likely to contribute to this morbidity and mortality.

People living with mental ill health have low vaccination rates. Studies in clinical cohorts with severe mental illness have found influenza vaccination rates from 25% (Lorenz *et al.*, 2013) to as low as 7% (Young *et al.*, 2015), and rates for vaccination against pneumococcal pneumonia, measles and hepatitis that are below 10% (Miles *et al.*, 2020). US veterans with mental health conditions are up to twenty percent less likely than other veterans to receive vaccination for pneumococcal disease or influenza (Druss *et al.*, 2002). In Israel, vaccination rates against COVID-19 were 25% lower in people with non-affective psychoses than in the broader population (Goldberger *et al.*, 2022).

Rates of vaccine-preventable conditions are also increased in people living with mental ill health. Recent meta-analyses found that people with serious mental illness had up to five-fold increases in the prevalence of Hepatitis B (Ayano *et al.*, 2018, Hughes *et al.*, 2016), with higher rates in men (Ayano *et al.*, 2018) and in people with a diagnosis of schizophrenia (Lluch and Miller, 2019). Fewer studies have examined other vaccine-preventable conditions. In Taiwan's national claims database people with a broad range of mental health conditions were 30% more likely to be diagnosed with Herpes Zoster (Yang *et al.*, 2011), and people diagnosed with bipolar disorder were more than twice as likely to be diagnosed with pneumonia or tuberculosis (Chen *et al.*, 2022). A population study from Israel found that people with a mental health condition were less likely to be diagnosed with COVID-19, but this may have reflected lower testing rates (Goldberger *et al.*, 2022).

Few studies have examined hospitalisations or deaths due to vaccine-preventable illnesses in large, representative populations of people with a mental health condition. Studies from England, Sweden and Denmark have suggested a two to three times increased risk of hospitalisation for influenza and pneumonia in mental health service users, even when controlling for confounders such as smoking status (Crump *et al.*, 2013a, Crump *et al.*, 2013b, Davydow *et al.*, 2016, Seminog and Goldacre, 2013). In Israel, people with mental health diagnoses had more than twice the population risk of hospitalisation or death after diagnosis with COVID-19 (Goldberger *et al.*, 2022). Two Australian studies have examined sub-national (state) populations, finding a two- to four-fold increased risk of hospitalisation for vaccine-preventable conditions including influenza, pneumonia and hepatitis (Mai *et al.*, 2011, Sara *et al.*, 2021).

More evidence about vaccine-preventable hospitalisations is required to understand the scale of this issue and to identify priority areas or groups for action. Most studies have focused on hospitalisations for respiratory illness. No study has reported on a broad range of specific vaccine-preventable conditions or presented age-specific risks. Many of the studies above include data from the 1960s to the early 2000s, however we need updated data because of new vaccine approaches and new patterns of illness, including COVID 19.

The current study describes hospitalisation rates for vaccine-preventable conditions in the state of New South Wales (NSW), Australia. We examine hospitalisation rates over five years for nineteen specific vaccine-preventable conditions. We compare rates for the overall adult population to those in mental health service users, and report age- and sex-specific rates adjusting for differences in socio-economic disadvantage.

Methods

Study design

All vaccine-preventable hospitalisations (VPH) were identified over five years in the state of New South Wales (NSW), Australia using retrospective observational methods. The outcome of interest was the number of vaccine-preventable hospitalisations (episodes of hospital care) (VPH) and the number of VPH related hospital days, expressed as rates per 100,000 population per year.

Data linkage

Data from NSW public hospitals, private hospitals, public community mental health services and the NSW Register of Births Deaths and Marriages were linked by the NSW Centre for Health Record Linkage (CHeReL), using -probabilistic record linkage based on individuals' names, date of birth, addresses and health service identifiers. The linkage process is designed to give a false positive linkage rate of around 5 per 1000 records. More detail on the datasets and linkage methods is provided elsewhere (Sara *et al.*, 2019)

Study setting

Vaccine-preventable hospitalisations to NSW private and government-funded (public) hospitals were examined. Around 60% of Australian hospital care is provided through state operated public hospitals (Australian Institute of Health and Welfare, 2022a). Private hospitals mainly provide non-emergency care for individuals opting-in to private health insurance. Hospital diagnoses are recorded using the International Classification of Diseases, 10th Edition, Australian Modification (National Centre for Classification in Health, 2010).

Mental health service users were defined by receiving any mental health care from a NSW public or private hospital, or a state government operated (public) community mental health services. Public community mental health services provide care to around 2% of the NSW population each year (Australian Institute of Health and Welfare, 2022b), focusing on emergency care and longer term care of severe or complex disorders. Primary and private mental health outpatient services see around 11% of the NSW population each year (Australian Institute of Health and Welfare, 2022b), and focus mainly on higher prevalence mental health conditions: data for those services are not included in the current linkage.

Primary outcome: Vaccine-preventable hospitalisations

The main outcome was admission to any NSW public or private hospital with a primary or secondary diagnosis of a vaccine-preventable hospitalisation (VPH) during a five-year period from 1 July 2015 to 30 June 2020. We examined 19 vaccine-preventable conditions, using specifications of the Australian Commission on Quality and Safety in Health Care (Australian Commission on Safety and Quality in Health Care, 2017) supplemented with additional codes. These were divided into three subgroups: respiratory conditions (influenza, pneumococcal pneumonia, haemophilus pneumonia, pertussis, diphtheria, COVID-19); hepatitis B, and; other vaccine-preventable conditions (herpes zoster, tuberculosis, varicella, rotaviral enteritis, mumps, measles, haemophilus meningitis, tetanus, rubella, cholera, acute poliomyelitis). Hepatitis B was grouped separately because of its high prevalence and because its mode of transmission and associations were assumed to be different from those of other conditions. Hepatitis C was not included as it is not currently vaccine preventable.

Mental health cohort definition

Mental health service users were defined as NSW residents aged 18-100 years who had any mental health hospitalisation or public community mental health contact between 1 July 2013 and 30 June 2020. Mental health hospitalisations were defined as episodes of care in a public or private hospital with a primary diagnosis of a non-organic mental health condition (ICD-10 codes F10-F99) or at least one day in a designated mental health unit. Community contacts included face-to-face or telehealth contacts with NSW public community mental health services. Services to non-NSW residents, administrative contacts, case conferences and contacts by community teams with hospital inpatients were excluded. Sex and area of residence were defined at the first (index) contact in the observation period. Age was estimated at the midpoint of the study period. Mental health status was treated as time-dependent, with events (hospitalisations) and exposure time (person years) calculated from the date of each person's index mental health contact in the study period, or from the start of the study period (1 Jul 2015) if the index contact occurred during the two-year pre-study look-back period. Deaths during the study period were identified by linkage to the NSW Register of

Births, Deaths and Marriages. If death occurred during the study period, time from the date of death to the study end was excluded when calculating hospitalisation rates.

Data analysis

Data assembly and standardisation were conducted in SAS Enterprise Guide v7.15 and SAS v9.4. VPH rates per 100,000 person-years were calculated for Mental Health (MH) and non-MH groups. VPH episode and day rates were calculated for (i) any VPH, (ii) three VPH groups (respiratory, hepatitis B, other), and (iii) 19 individual VPH conditions. Episodes with multiple VPH diagnoses were counted separately for condition-specific rates but treated as a single episode when calculating overall VPH rates.

For calculation of standardised rates, ~~Age-specific~~ admission rates were first calculated separately for each study group, subgroup, vaccine preventable condition group, individual vaccine preventable condition, and stratum of age ~~using eight age-bands~~ (18-24,25-34,35-44,45-54,55-64,65-75,75-84,85+), sex and quintile of socioeconomic disadavantage. Socioeconomic disadvantage was estimated from the person's area of residence, using the Australian Bureau of Statistics Index of Relative Socioeconomic Disadvantage (IRSD) (Australian Bureau of Statistics, 2006). This index is calculated for Australian geographical areas using 17 census-derived variables measuring income, government welfare support, education, home ownership, employment, household structure and English language proficiency. Denominators for rate calculations were (i) for mental health service users: stratum-specific populations from the mental health cohort and (ii) for other NSW residents: census-derived population estimates from the Australian Bureau of Statistics for the midpoint of the study period, after subtracting the relevant mental health service user population. Adjusted Incidence Rate Ratios (aIRRs) and 95% Log Normal confidence intervals were then calculated ~~after~~ by direct standardisation for age, sex and socioeconomic status ~~compared to the NSW population~~, using the SAS procedure "Proc STDRATE" (SAS Institute Inc, 2020). Standardised rates were not calculated where the number of VPH events in the MH cohort was less than 20.

A supplementary analysis was conducted to examine admission rates for COVID-19, using public hospital data from July 2020 to September 2021. The original planned analysis included all NSW public and private hospitals, however private hospital data were available only to June 2020. That covered three months of the first wave of the NSW pandemic (April to June 2020), during which hospital admissions for COVID-19 were uncommon. Public hospital data were available to September 2021, which included the peak of the much larger NSW COVID-19 Delta wave. Rates and adjusted rate ratios were calculated using the same methods and population denominators as the main analyses.

Two ~~A~~ subgroup analyses~~is~~ ~~was~~ were conducted. First we ~~to~~ examined VPH rates in mental health service users with severe or persistent mental illness (SPMI), who were defined as people with (i) any diagnosis of schizophrenia, schizoaffective disorder, bipolar disorder or psychotic depression, or (ii) more than two years of contact with mental health services, as measured from the dates of their first and last mental health service contacts during the observation and pre-study lookback periods. Second, we examined VPH rates in mental health service users who had received hospital care, compared to people whose only mental health service contact was with community-based, non-admitted mental health services.

Ethics approval

The study was approved by the NSW Population and Health Service Research Ethics Committee (HREC/17/CIPHS/48. CINSW Refs 2017/HRE1105, 2019/UMB0208), and the Aboriginal Health and Medical Research Council of NSW (Ref 1564/19).

Results

We identified 500,548 people who had at least one in-scope contact with NSW mental health services in the study or look-back periods. Of these, we excluded 62,514 (12.5%) people aged under 18 at first contact, 12,987 (2.6%) with no valid age recorded, and 72 (0.01%) with sex missing or recorded as “other than male or female”. We also excluded 5,706 people (1.1%) who had contact in the pre-study look-back period but died before the start of the observation period. After these exclusions 418,915 mental health service users were included in calculation of standardised rates. Nineteen percent had a diagnosis of a psychotic disorder recorded, and approximately half (51%) received community mental health care only (see Supplementary Table 1). Compared to NSW population estimates for the mid-point of the study period (Jan 2018), mental health service users were more likely to be female, aged under 45, and to live in regions in the most disadvantaged two quintiles of the NSW population (Table 1).

During the study period there were 94,180 vaccine-preventable hospitalisations (VPH) to NSW public or private hospitals (Table 2). Nearly all (89%) occurred in public hospitals. These hospitalisations included 97,910 individual vaccine-preventable condition diagnoses, because a small number of admissions included more than one such diagnosis. The most frequently diagnosed individual conditions were influenza (41% of all VPH diagnoses), hepatitis B (33%) and herpes zoster (10%). These three conditions, along with two vaccine-preventable pneumonias (haemophilus pneumoniae, streptococcus pneumoniae) made up 98% of all VPH diagnoses. The study period for the primary analysis included only the first three months of the COVID-19 pandemic; 668 COVID admissions were recorded, including 27 in mental health service users. Conditions with fewer than 20

admissions in the mental health cohort are not reported separately but have been included in group and state totals. These include mumps (109 total admissions), measles (30), haemophilus meningitis (24), tetanus (16), rubella (12), cholera (10), diphtheria (9) and acute poliomyelitis (9).

Compared to other NSW residents, mental health service users were 2.6 times more likely to experience a vaccine-preventable hospitalisation, with an aIRR of 3.2 (95% CI 2.1 – 2.2) after standardising for age, sex and socioeconomic disadvantage. Relative risk was increased for all VPH types other than COVID-19, with the highest relative risk for hepatitis B (aIRR 4.4, 95% CI 4.3 – 4.6).

A supplementary analysis was conducted for the period from July 2020 to September 2021 to examine hospitalisation rates for COVID, using data for NSW public hospitals only (Table 3). During this period there were 10,186 admissions for COVID-19 to NSW public hospital and "hospital in the home" services (1,243 in mental health service users, 8,943 in other NSW residents). Overall VPH admission rates fell during this period, dropping by 21% in the broader population and 12% in mental health service users, with substantial reductions in admission for most VPH types and almost complete absence of influenza hospitalisations. In this period, mental health users were twice as likely to have a hospital admission with a COVID-19 diagnosis (aIRR 2.0, 95% CI 1.9 – 2.2).

Patterns of vaccine-preventable hospitalisation differed by age, sex and type of vaccine-preventable condition (Figure 1 and Supplementary Table ~~1~~2). In the broader NSW population VPH rates approximately doubled for each decade over age 55. In mental health service users, rates increased from a younger age with the highest relative risks in mental health service users aged 35-64. Mental health service users aged 35-54 had very high relative risks of admission for hepatitis B. In mental health service users aged 75 and above, relative risks converged towards population rates.

Earlier age-related increases in risk of VPH admission were also reflected in younger average age at admission for mental health service users. On average mental health service users were 9 years younger than other NSW residents when admitted with a VPH diagnosis (Figure 2). This age gap was largest for respiratory conditions, particularly for pneumonia due to haemophilus (13 years) and streptococcus (11 years).

Examining the secondary outcome of hospital bed days, mental health service users had a slightly longer average length of hospital stay for vaccine-preventable conditions (MH service users 9.7 days, other NSW residents 8.1 days). Because of the combination of a longer length of stay and a higher hospitalisation rate, mental health service users experienced 3.8 times more hospital bed days per capita than other NSW residents (95% CI 3.77 – 3.82) after adjusting for age, sex and socioeconomic status (Supplementary Table 2).

In subgroup analysis, people with severe or persistent mental illness (SPMI) made up 33% of people and 40% of person-years in the mental health cohort (Supplementary Table 34). Standardised VPH rates were highest in the SPMI group (aIRR 4.0, 95% CI 3.9-4.1), but also significantly elevated in other mental health service users (aIRR 2.8, 95% CI 2.8-2.9) compared to other NSW residents. The highest relative risk in the SPMI group was for Hepatitis B (aIRR 6.1, 96% CI 5.8 - 6.3). Both groups of mental health service users had significantly increased risk of hospitalisation for all disorders examined. The SPMI subgroup had significantly higher standardised VPH rates than the other mental health subgroup overall, for hepatitis and respiratory conditions. However, the other mental health subgroup had higher standardised VPH rates than the SPMI subgroup for other vaccine-preventable conditions, including herpes zoster, tuberculosis and varicella. A similar pattern was seen in subgroup analysis based on the type of mental health care received (Supplementary Table 5). Compared to non-mental health-service users, the relative risk of any VPH was slightly higher in people with any mental health hospital care (aIRR 3.6, 95% CI3.5-3.7) than in people receiving community mental health care only (aIRR 3.1, 95% CI 3.0 – 3.2). Compared to people with community mental health care only, people with admitted mental health care had slightly higher risk of VPH for hepatitis and respiratory conditions but slightly lower risk for other vaccine-preventable conditions.

Discussion

We examined hospitalisations for 19 individual vaccine-preventable conditions in 418,915 adult mental health service users over a five-year period and compared hospitalisation rates to a broader population of 6.2 million adults. Each year in NSW there were nearly 20,000 VPH, occupying more than 120,000 bed days. After adjusting for differences in age, sex and socioeconomic disadvantage, mental health service users were 3.2 times more likely than other NSW residents to be admitted to hospital for a vaccine-preventable condition and had 3.8 times more days in hospital per capita for those conditions. The highest relative risk in mental health service users was for hepatitis B, but VPH rates were increased across the spectrum of vaccine-preventable conditions, including COVID-19. The rate of VPH was slightly higher in the subgroup of mental health service users with severe and persistent mental illness, but other mental health service users also had significantly increased rates.

Previous studies have found that people with prior mental health service contact were two to three times more likely to be admitted for influenza and pneumonia (Crump *et al.*, 2013a, Mai *et al.*, 2011, Seminog and Goldacre, 2013). We found a 2.8 fold increase in admission rate for respiratory conditions, similar to findings of Crump (Crump *et al.*, 2013b) and Davydow (Davydow *et al.*, 2016). We also found similar increases for other respiratory conditions including pertussis, as well as for

herpes zoster and varicella. Our main analysis covered only the first few months of the COVID-19 pandemic in NSW and did not show increased hospitalisation for COVID-19. However secondary analysis of a longer period of public hospital data found a two-fold risk of COVID admission in mental health service users, consistent with recent findings from Israel (Goldberger *et al.*, 2022). These findings of increased hospitalisation rates across a broad spectrum of conditions suggest that causes may also be broad, and that prevention strategies may need to consider diverse conditions and risk groups.

We found that mental health service users had a mean age that was almost a decade younger when admitted for vaccine-preventable conditions, and that those in their 40s and 50s had particularly elevated risk of admission for vaccine-preventable respiratory conditions. The normal age-related risk curve for vaccine-preventable hospitalisations appears to be shifted to the left ~~left~~ in mental health service users. For effective prevention, public health strategies may need to consider the earlier onset of chronic medical illness in this group (Firth *et al.*, 2019). In Australia, as in many countries, mental health conditions are not currently considered amongst criteria for ~~earlier~~ subsidised access to vaccination at a younger age than the general public for conditions such as influenza, pneumococcal pneumonia or herpes zoster (Australian Technical Advisory Group on Immunisation, 2022). Hepatitis B vaccines are only subsidised for those under 20 years old or for refugee backgrounds, and Influenza vaccines are subsidised for those over 65 years old, Aboriginal and Torres Strait Islander people (Australian Technical Advisory Group on Immunisation, 2022), creating a large barrier to both service providers and individuals in increasing adult vaccination for this population. ~~.~~ The influenza vaccine is subsidised in Australia for people with a medical condition that puts them at higher risk of getting serious influenza disease, however severe mental illness is not included in this list (Australian Technical Advisory Group on Immunisation, 2022). By contrast, in New Zealand free access to influenza vaccines has ~~recently~~ been expanded to include people living with a severe mental illness or accessing a mental health service (Manatu Hauora New Zealand Ministry of Health, 2022).

Our findings suggest that vaccine-preventable illnesses cause significant harm in people living with mental health conditions, and that increased hospitalisation risks are not simply explained by differences in age, sex or socioeconomic status. More study is needed to demonstrate the mechanisms of increased vaccine-preventable hospitalisations in this group. We do not currently have data on vaccination status to explore these interactions in our study population.

We speculate that increased hospitalisations arise through interactions between increased exposure, reduced vaccination, more severe illness, greater medical comorbidity and other risk

factors. Therefore improving vaccination rates is likely to be an important but not sufficient strategy for reducing vaccine-preventable harms in mental health service users. We need strategies to reduce policy barriers, increase access and improve vaccination coverage. We also need to understand and address the many other risk factors likely to also contribute to the development of more severe vaccine-preventable conditions in this group.

-Increased rates of Hepatitis B in people with severe mental illness may reflect greater exposure through injecting drug use (Hughes *et al.*, 2016, Lluch and Miller, 2019), and there is the potential that thefor many people exposure to hepatitis B exposure may have occurred prior to the development of mental illness., Additionally and, this group may also have greater exposure to respiratory infections due to housing conditions or exposures in health-care settings. Low vaccination rates are likely to contribute to high rates of admission for respiratory conditions (Druss *et al.*, 2002, Miles *et al.*, 2020), but no studies have reported both vaccination rates and harms in the same individuals. People living with mental health conditions may have other risk factors and comorbidities causing more severe illness once exposed to infection (Firth *et al.*, 2019). For example, smoking rates are increased in people living with mental health conditions, and smoking is a risk factor for more severe illness and ICU admission following respiratory infections, including COVID (Vardavas and Nikitara, 2020). However, some studies have found that increased hospitalisation risk for respiratory illness persists after adjusting for smoking status and comorbid medical or substance use conditions (Crump *et al.*, 2013a, Crump *et al.*, 2013b, Davydow *et al.*, 2016, Mai *et al.*, 2011), suggesting that other factors also contribute. The longer length of stay for VPH admissions in our study may reflect more severe illness, social or other barriers to discharge, or a combination of these factors. There has also been speculation that psychological distress or depressive symptoms may reduce the immune response to vaccination, increasing the risk of illness even when individuals are vaccinated (Abdeljaber *et al.*, 1994, Ford *et al.*, 2019, Glaser *et al.*, 2003, Segerstrom *et al.*, 2012, Wang *et al.*, 2016).

Strengths and limitations

This study identifies all vaccine-preventable hospital admissions for a population of more than 6 million adults and examines a population-wide cohort of people with mental health conditions. This allows a whole population view of a wide range of specific vaccine-preventable conditions. However there are several limitations to the datasets used in this study.

First, we do not currently have linked data on vaccination status, and so cannot examine the relationship between vaccination status and increased hospitalisation rates.

Second, Oour dataset does not include community mental health service contacts with primary care or private mental health providers. Our cohort was defined using hospital and public community mental health data, and is likely to represent people with more severe or longstanding illness sufficient to require hospital admission or be treated by public community services. They may not be representative of broader groups receiving care only in primary care or private settings for conditions such as depression or anxiety.

Third, our dataset does not include medication prescription data, and as such we were unable to analyse if the use of psychotropic medications was associated with vaccine preventable hospitalizations. More severe infectious disease in people living with mental health conditions is likely to reflect many causes including shared risk factors, delayed care, or biological factors such as impaired immune responses. A recent meta-analysis found that exposure to a wide range of psychotropics was also associated with increased COVID-related mortality (Vai *et al.*, 2021). However, this may reflect risk associated with the conditions for which those medications were prescribed, rather than independent effects of the medications. Studies controlling for likely clinical and demographic confounders have reported a more complex relationship between medications and COVID susceptibility or severity. In people with severe mental illness mood stabilisers were associated with increased risk of COVID infection, while Clozapine and Paliperidone were associated with reduced risk. In a large emergency and acute care cohort, (Oskotsky *et al.*, 2021) prior prescription of a selective serotonin reuptake inhibitor was associated with reduced risk of COVID mortality after adjusting for demographic, clinical, co-morbidity and treatment setting variables. More study is needed to understand possible harmful or protective effects of psychotropic medications on vaccine-preventable illnesses (Nemani *et al.*, 2021).

Fourth, Cvommunity mental health records in our large administrative dataset are often missing diagnostic information. We therefore combined diagnosis and duration of service contact to define a subgroup with severe or persistent mental illness. This is a common approach in operational definitions of serious mental illness (Gonzales *et al.*, 2022). However, this causes imprecision in our subgroup analyses because some people not meeting the operational definition of severe and persistent mental illness may still have had significant risk or impairment.

Finally, Oour current dataset does not cover the significant COVID-19 Omicron variant wave, which in NSW peaked in the last quarter of 2021 and early 2022. Within the NSW health system, models of care and patterns of hospitalisation for COVID continue to evolve. We plan to revise our current estimates for COVID as further data become available in future linkage cycles. We do not currently

~~have linked data on vaccination status, and so cannot examine the relationship between vaccination status and increased hospitalisation rates.~~

Conclusions

COVID-19 reminds us that vaccine programs are essential in reducing the impact of preventable infections on individuals and health systems. There have been recent calls to ensure that people living with mental illness are prioritised in the development of COVID vaccination strategies (De Hert *et al.*, 2020) and have equitable access to all vaccination programmes (Equally Well Alliance, 2021).

We need ongoing study of the mechanisms and impacts of vaccine-preventable harms in people living with mental illness, to identify priority conditions and groups, and to evaluate effective models for intervention.

References

Abdeljaber, M. H., Nair, M. P. N., Schork, M. A. & Schwartz, S. A. (1994). Depressed Natural Killer Cell Activity in Schizophrenic Patients. *Immunological Investigations* **23**, 259-268.

Australian Bureau of Statistics (2006). *An Introduction to Socio-Economic Indexes for Areas (SEIFA). 2093.0*. Commonwealth of Australia: Canberra.

Australian Commission on Safety and Quality in Health Care (2017). *A guide to the potentially preventible hospitalisations indicator in Australia* Australian Commission on Safety and Quality in Health Care: Sydney.

Australian Institute of Health and Welfare (2022a). Hospital Activity. In *Australian Hospital Statistics*. AIHW: Canberra.

Australian Institute of Health and Welfare (2022b). Mental Health Services in Australia: National Healthcare Agreement Indicators (Table NHA 17.1) AIHW: Canberra.

Australian Technical Advisory Group on Immunisation (2022). Australian Immunisation Handbook. Department of Health and Aged Care, Australian Government: Canberra.

Ayano, G., Tulu, M., Haile, K., Assefa, D., Habtamu, Y., Araya, G. & Yohannis, Z. (2018). A systematic review and meta-analysis of gender difference in epidemiology of HIV, hepatitis B, and hepatitis C infections in people with severe mental illness. *Annals of General Psychiatry* **17**.

Chen, P. H., Tsai, S. Y., Pan, C. H., Chen, Y. L., Su, S. S., Chen, C. C. & Kuo, C. J. (2022). Prevalence and 5-year trend of incidence for medical illnesses after the diagnosis of bipolar disorder: A nationwide cohort study. *Aust N Z J Psychiatry* **56**, 1164-1176.

Crump, C., Sundquist, K., Winkleby, M. A. & Sundquist, J. (2013a). Comorbidities and Mortality in Bipolar Disorder: A Swedish National Cohort Study. *JAMA Psychiatry* **70**, 931-939.

Crump, C., Winkleby, M. A., Sundquist, K. & Sundquist, J. (2013b). Comorbidities and mortality in persons with schizophrenia: a Swedish national cohort study. *Am J Psychiatry* **170**, 324-33.

Davydow, D. S., Ribe, A. R., Pedersen, H. S., Fenger-Gron, M., Cerimele, J. M., Vedsted, P. & Vestergaard, M. (2016). Serious Mental Illness and Risk for Hospitalizations and Rehospitalizations for Ambulatory Care-sensitive Conditions in Denmark: A Nationwide Population-based Cohort Study. *Med Care* **54**, 90-7.

De Hert, M., Mazereel, V., Detraux, J. & Van Assche, K. (2020). Prioritizing COVID-19 vaccination for people with severe mental illness. *World Psychiatry* **Preprint**.

Druss, B. G., Rosenheck, R. A., Desai, M. M. & Perlin, J. B. (2002). Quality of preventive medical care for patients with mental disorders. *Med Care* **40**, 129-36.

Equally Well Alliance (2021). Fair and equitable access to vaccination for people living with mental illness and substance use disorders: a global call to action.

Firth, J., Siddiqi, N., Koyanagi, A., Siskind, D., Rosenbaum, S., Galletly, C., Allan, S., Caneo, C., Carney, R., Carvalho, A. F., Chatterton, M. L., Correll, C. U., Curtis, J., Gaughran, F., Heald, A., Hoare, E., Jackson, S. E., Kisely, S., Lovell, K., Maj, M., McGorry, P. D., Mihalopoulos, C., Myles, H., O'Donoghue, B., Pillinger, T., Sarris, J., Schuch, F. B., Shiers, D., Smith, L., Solmi, M., Suetani, S., Taylor, J., Teasdale, S. B., Thornicroft, G., Torous, J., Usherwood, T., Vancampfort, D., Veronese, N., Ward, P. B., Yung, A. R., Killackey, E. & Stubbs, B. (2019). The Lancet Psychiatry Commission: a blueprint for protecting physical health in people with mental illness. *The Lancet Psychiatry* **6**, 675-712.

Ford, B. N., Yolken, R. H., Dickerson, F. B., Teague, T. K., Irwin, M. R., Paulus, M. P. & Savitz, J. (2019). Reduced immunity to measles in adults with major depressive disorder. *Psychol Med* 49, 243-249.

Glaser, R., Robles, T. F., Sheridan, J., Malarkey, W. B. & Kiecolt-Glaser, J. K. (2003). Mild depressive symptoms are associated with amplified and prolonged inflammatory responses after influenza virus vaccination in older adults. *Arch Gen Psychiatry* 60, 1009-14.

Goldberger, N., Bergman-Levy, T., Haklai, Z., Yoffe, R., Davidson, M., Susser, E., Levi, L., Elhasid, T. & Weiser, M. (2022). COVID-19 and severe mental illness in Israel: testing, infection, hospitalization, mortality and vaccination rates in a countrywide study. *Mol Psychiatry*.

Gonzales, L., Kois, L. E., Chen, C., Lopez-Aybar, L., McCullough, B. & McLaughlin, K. J. (2022). Reliability of the Term "Serious Mental Illness": A Systematic Review. *Psychiatr Serv* 0, appips202100661.

Hughes, E., Bassi, S., Gilbody, S., Bland, M. & Martin, F. (2016). Prevalence of HIV, hepatitis B, and hepatitis C in people with severe mental illness: a systematic review and meta-analysis. *The Lancet Psychiatry* 3, 40-48.

Lluch, E. & Miller, B. J. (2019). Rates of hepatitis B and C in patients with schizophrenia: A meta-analysis. *Gen Hosp Psychiatry* 61, 41-46.

Lorenz, R. A., Norris, M. M., Norton, L. C. & Westrick, S. C. (2013). Factors associated with influenza vaccination decisions among patients with mental illness. *Int J Psychiatry Med* 46, 1-13.

Mai, Q., Holman, C. D., Sanfilippo, F. M. & Emery, J. D. (2011). The impact of mental illness on potentially preventable hospitalisations: a population-based cohort study. *BMC Psychiatry* 11, 163.

Manatu Hauora New Zealand Ministry of Health (2022). People with serious mental health or addiction needs. New Zealand Government: Wellington.

Miles, L. W., Williams, N., Luthy, K. E. & Eden, L. (2020). Adult Vaccination Rates in the Mentally Ill Population: An Outpatient Improvement Project. *J Am Psychiatr Nurses Assoc* 26, 172-180.

National Centre for Classification in Health (2010). *The International statistical classification of diseases and related health problems, tenth revision, Australian modification (7th edn)*. National Centre for Classification in Health, Faculty of Health Sciences, The University of Sydney: Sydney.

Nemani, K., Li, C., Olfson, M., Blessing, E. M., Razavian, N., Chen, J., Petkova, E. & Goff, D. C. (2021). Association of Psychiatric Disorders With Mortality Among Patients With COVID-19. *JAMA Psychiatry* 78, 380-386.

Oskotsky, T., Maric, I., Tang, A., Oskotsky, B., Wong, R. J., Aghaeepour, N., Sirota, M. & Stevenson, D. K. (2021). Mortality Risk Among Patients With COVID-19 Prescribed Selective Serotonin Reuptake Inhibitor Antidepressants. *JAMA Netw Open* 4, e2133090.

Sara, G., Arumuganathan, M., Chen, W., Wu, F., Currow, D., Large, M., Mulder, C., Ramanuj, P. P. & Burgess, P. M. (2019). Cohort profile: Mental Health Living Longer: a population-wide data linkage to understand and reduce premature mortality in mental health service users in New South Wales, Australia. *BMJ Open* 9, e033588.

Sara, G., Chen, W., Large, M., Ramanuj, P., Curtis, J., McMillan, F., Mulder, C. L., Currow, D. & Burgess, P. (2021). Potentially preventable hospitalisations for physical health conditions in community mental health service users: a population-wide linkage study. *Epidemiol Psychiatr Sci* 30, e22.

SAS Institute Inc (2020). The STDRATE Procedure. In *SAS/STAT® 15.2 User's Guide*. SAS Institute Inc.

Segerstrom, S. C., Hardy, J. K., Evans, D. R. & Greenberg, R. N. (2012). Vulnerability, distress, and immune response to vaccination in older adults. *Brain Behav Immun* 26, 747-53.

Seminog, O. O. & Goldacre, M. J. (2013). Risk of pneumonia and pneumococcal disease in people with severe mental illness: English record linkage studies. *Thorax* 68, 171-6.

Vai, B., Mazza, M. G., Delli Colli, C., Foiselle, M., Allen, B., Benedetti, F., Borsini, A., Casanova Dias, M., Tamouza, R., Leboyer, M., Benros, M. E., Branchi, I., Fusar-Poli, P. & De Picker, L. J. (2021). Mental disorders and risk of COVID-19-related mortality, hospitalisation, and intensive care unit admission: a systematic review and meta-analysis. *The Lancet Psychiatry* doi 10.1016/s2215-0366(21)00232-7.

Vardavas, C. I. & Nikitara, K. (2020). COVID-19 and smoking: A systematic review of the evidence. *Tob Induc Dis* 18, 20.

Wang, Y., Yu, L., Zhou, H., Zhou, Z., Zhu, H., Li, Y., Zheng, Z., Li, X. & Dong, C. (2016). Serologic and molecular characteristics of hepatitis B virus infection in vaccinated schizophrenia patients in China. *J Infect Dev Ctries* 10, 427-31.

Yang, Y. W., Chen, Y. H. & Lin, H. W. (2011). Risk of herpes zoster among patients with psychiatric diseases: a population-based study. *Journal of the European Academy of Dermatology & Venereology* 25, 447-53.

Young, S., Dosani, N., Whisler, A. & Hwang, S. (2015). Influenza Vaccination Rates Among Homeless Adults With Mental Illness in Toronto. *Journal of Primary Care & Community Health* 6, 211-4.

Table 1 Cohort description

	MH service users n (% of group)	NSW Population aged 18+ n (% of group)
**Total**	418,915 (100%)	6,162,602 (100%)
**Sex**		
Female	217,843 (52%)	3,137,557 (51%)
Male	201,072 (48%)	3,025,046 (49%)
**Age group**		
18-24	70,920 (17%)	733,781 (12%)
25-34	87,513 (21%)	1,186,254 (19%)
35-44	80,886 (19%)	1,050,450 (17%)
45-54	66,529 (16%)	1,001,947 (16%)
55-64	46,695 (11%)	923,220 (15%)
65-74	30,765 (7%)	708,857 (12%)
75-84	21,244 (5%)	387,496 (6%)
85+	14,363 (3%)	170,598 (3%)
**Disadvantage quintile**		
Q1 Most	70,165 (17%)	1,124,329 (18%)
Q2	116,543 (28%)	1,078,653 (18%)
Q3	76,946 (18%)	1,305,848 (21%)
Q4	68,601 (16%)	1,240,146 (20%)
Q5 Least	71,433 (17%)	1,413,626 (23%)
Unknown	15,227 (4%)	-

Notes: Mental health service users aged 18-100, and NSW adult population, July 2015 to June 2020. Estimated NSW adult population at study midpoint (Jan 2018). Disadvantage quintiles based on person's address of residence, using Index of Relative Socioeconomic Disadvantage (IRSD).

TABLE 2: Vaccine-preventable hospitalisations (VPH) of adults (aged 18-100) to NSW public and private hospitals, July 2015 to June 2020, comparing people receiving any hospital or community mental health care to the rest of the NSW population. Adjusted incidence rate ratios (aIRR) after standardisation by (1) age and sex, (2) age, sex and socioeconomic disadvantage.

	VPH episodes (n)		Crude rate (per 100k person years)			Adjusted incidence rate ratios (aIRR), (95% CI)	
	MH care	No MH care	MH care	No MH care	IRR	1. Age, Sex.	2. Age, Sex, Disadvantage
**Any Vaccine-preventable**	**10,679**	**83,501**	**726.7**	**284.6**	**2.6**	**3.1 (3.0 - 3.1)**	**3.2 (3.1 - 3.3)**
**Hepatitis B**	**4,901**	**26,371**	**333.5**	**89.9**	**3.7**	**4.2 (4.1 - 4.4)**	**4.4 (4.3 - 4.6)**
**Respiratory infections (1)**	**4,918**	**45,820**	**334.7**	**156.1**	**2.1**	**2.7 (2.6 - 2.7)**	**2.8 (2.7 - 2.9)**
Influenza	3,609	35,381	245.6	120.6	2.0	2.5 (2.5 - 2.6)	2.7 (2.6 - 2.8)
Haemophilus pneumonia	505	4,618	34.4	15.7	2.2	2.7 (2.5 - 3.0)	2.8 (2.6 - 3.1)
Streptococcal pneumonia	901	6,220	61.3	21.2	2.9	3.5 (3.2 - 3.7)	3.6 (3.3 - 3.9)
COVID	27	641	1.8	2.2	0.8	1.0 (0.6 - 1.4)	0.9 (0.6 - 1.3)
Pertussis (whooping cough)	55	449	3.7	1.5	2.4	3.0 (2.2 - 4.0)	3.1 (2.3 - 4.2)
**Other (2)**	**1,029**	**12,535**	**70.0**	**42.7**	**1.6**	**2.1 (2.0 - 2.2)**	**2.2 (2.0 - 2.3)**
Herpes Zoster	761	9,106	51.8	31.0	1.7	2.2 (2.0 - 2.4)	2.3 (2.1 - 2.5)
Tuberculosis	162	2,301	11.0	7.8	1.4	1.6 (1.4 - 1.9)	1.8 (1.5 - 2.1)
Varicella (Chicken Pox)	101	1,108	6.9	3.8	1.8	2.2 (1.8 - 2.7)	2.1 (1.7 - 2.7)
Rotaviral enteritis	39	401	2.7	1.4	1.9	2.4 (1.7 - 3.3)	2.5 (1.7 - 3.5)

Notes: VPH: Vaccine-preventable Hospitalisation. MH: mental health. Disadvantage measured using index of relative socioeconomic disadvantage (IRSD) of address of residence. (1) Respiratory group total includes 9 hospitalisations for Diphtheria. (2) Other group total includes 22 hospitalisations for Mumps, Measles, Haemophilus meningitis, Tetanus, Rubella, Cholera, Acute poliomyelitis. The sum of individual conditions may exceed group totals because some admissions have more than one VPH diagnosis recorded.

Table 3: VPH admission rates, NSW public hospitals before and during COVID-19, by VPH condition and MH service user group.

	VPH episodes (n)		Rate (per 100k person years)			Adjusted Rate Ratio
PPH condition	MH service users	Other NSW residents	MH service users	Other NSW residents	IRR	aIRR (95% CI)
**July 2015 to June 2020**						
Total Vaccine-preventable	10,263	73,370	698.4	250.0	2.8	3.3 (3.3 - 3.4)
Hepatitis B	4,742	20,807	322.7	70.9	4.6	5.2 (5.0 - 5.3)
Respiratory infections	4,731	42,551	322.0	145.0	2.2	2.7 (2.7 - 2.8)
Influenza	3,456	32,742	235.2	111.6	2.1	2.6 (2.5 - 2.7)
Haemophilus pneumonia	487	4,280	33.1	14.6	2.3	2.8 (2.6 - 3.1)
Streptococcus pneumoniae	890	5,922	60.6	20.2	3.0	3.6 (3.3 - 3.9)
COVID	26	628	2.2	2.1	1.0	1.1 (0.8 - 1.7)
Other	957	11,195	65.1	38.2	1.7	2.2 (2.0 - 2.3)
Herpes Zoster	709	7,927	48.3	27.0	1.8	2.4 (2.2 - 2.6)
Tuberculosis	153	2,228	10.4	7.6	1.4	1.6 (1.3 - 1.9)
Varicella (Chicken Pox)	98	1,039	6.7	3.5	1.9	2.3 (1.8 - 2.8)
**July 2020 to September 2021 (includes COVID Delta wave)**						
Total Vaccine-preventable	2,662	16,556	550.7	221.2	2.5	2.7 (2.6 - 2.8)
Hepatitis B	988	4,495	204.4	60.1	3.4	3.9 (3.7 - 4.2)
Respiratory infections	1,466	10,030	303.3	134.0	2.3	2.2 (2.1 - 2.4)
Influenza	23	150	4.8	2.0	2.4	3.2 (2.0 - 5.1)
Haemophilus pneumonia	68	397	14.1	5.3	2.7	3.4 (2.6 - 4.5)
Streptococcus pneumoniae	155	642	32.1	8.6	3.7	4.3 (3.6 - 5.1)
COVID	1,243	8,943	257.1	119.5	2.2	2.0 (1.9 - 2.2)
Other	265	2,365	54.8	31.6	1.7	2.4 (2.1 - 2.7)
Herpes Zoster	218	1,786	45.1	23.9	1.9	2.7 (2.4 - 3.2)
Tuberculosis	30	432	6.2	5.8	1.1	1.1 (0.8 - 1.7)
Varicella (Chicken Pox)	50	282	10.3	3.8	2.7	3.4 (2.5 - 4.7)

Figure 1: Vaccine-preventable Hospitalisations (VPH) in NSW adults aged 18-100, by age group, gender and type of vaccine-preventable condition.

All Vaccine Preventable Conditions

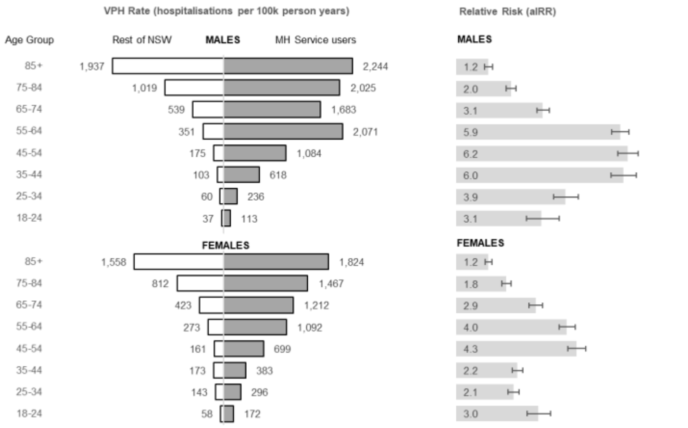

Hepatitis B

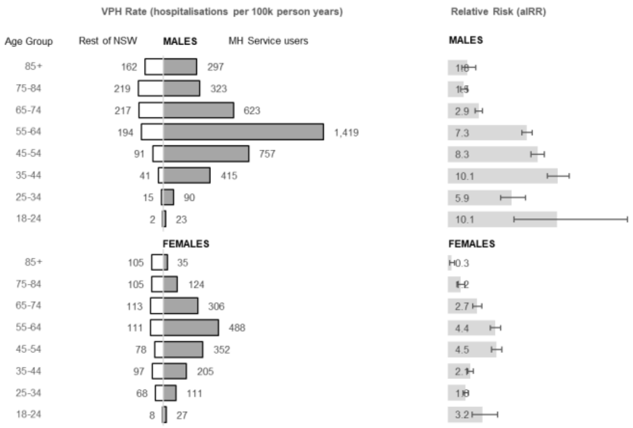

Respiratory Conditions

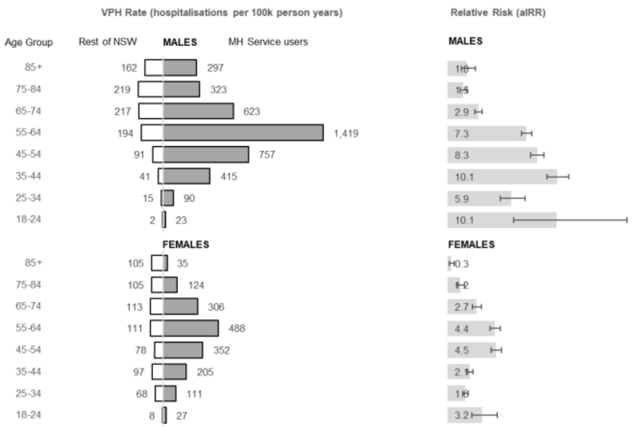

Other Vaccine Preventable Conditions

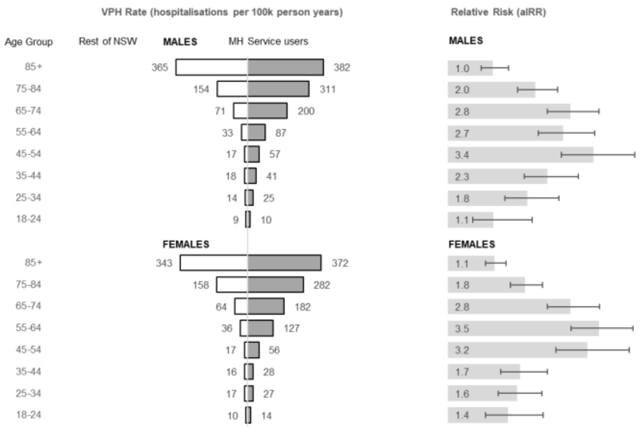

Figure 2: Average age at Vaccine-preventable Hospitalisation (VPH), comparing mental health (MH) service users to other NSW residents.

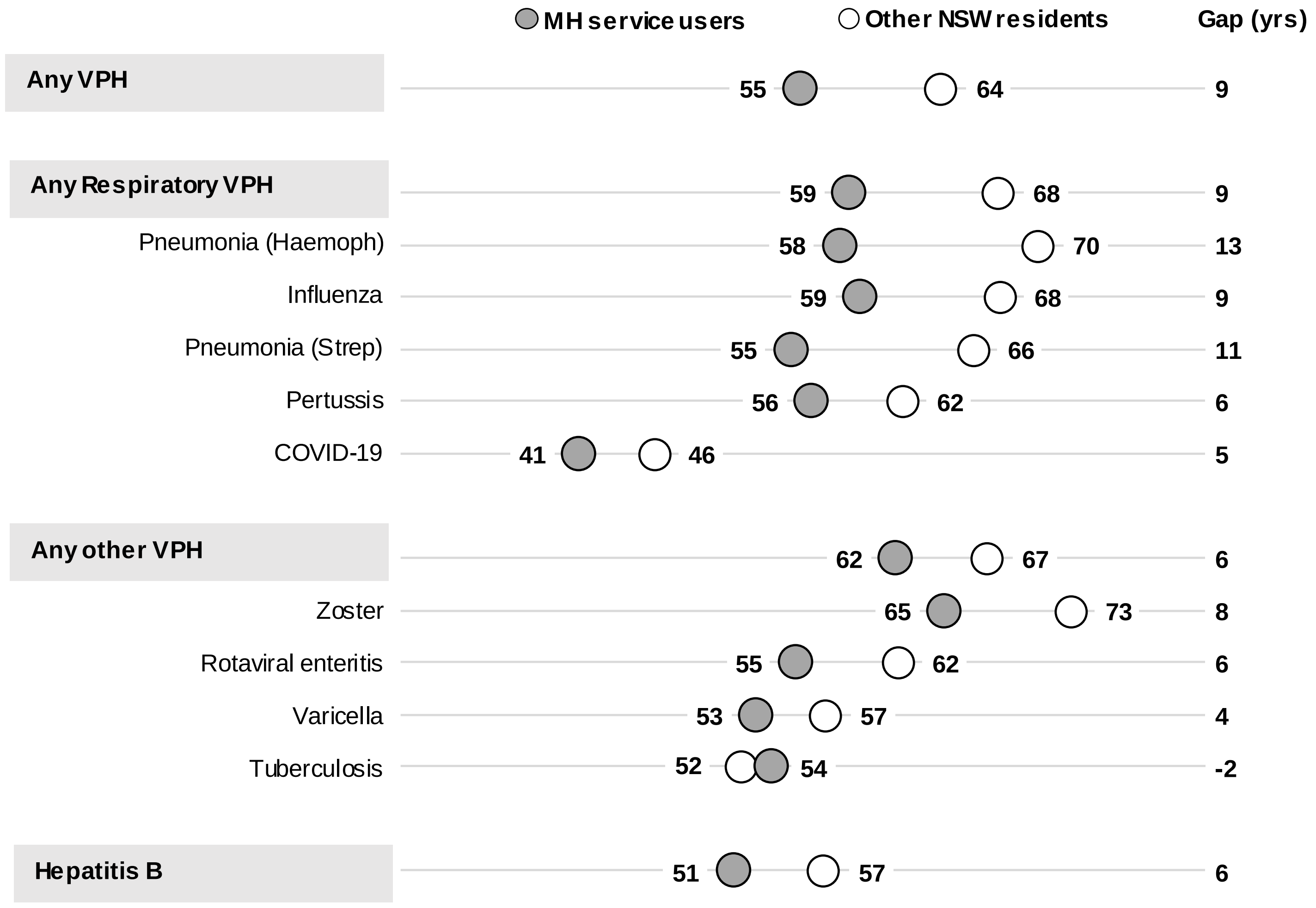

SUPPLEMENTARY MATERIAL

SUPPLEMENTARY TABLE 1 Additional data on diagnosis and characteristics of care, mental health cohort. Diagnoses were obtained from all service contacts for an individual in the study period. Diagnostic hierarchy was applied in the order listed in the table. "Unspecified MH diagnosis" is due to the high rate of diagnosis code 'F99' (Mental health diagnosis not otherwise specified) in people who only received community care (see methods). Severe or persistent mental illness defined as any diagnosis of psychosis or a duration of mental health care greater than two years.

**Characteristics**	**N**	**Pct**
**Total MH service users**	418,915	100%
**Diagnosis group**	-	-
Psychotic disorders	77,630	19%
Other specified MH diagnoses	196,393	47%
Self-harm or suicide attempt only	5,654	1.3%
Unspecified MH diagnosis	133,876	32%
Medical / non-mental health diagnosis only	5,266	1.3%
No diagnosis recorded	96	0.0%
**Type of mental health care**	-	-
Community MH care only	214,628	51%
Community and hospital MH care	140,102	33%
Hospital MH care only	64,185	15%
**Type of mental health hospital care**	-	-
No MH hospital care	214,628	51%
Public hospital only	164,550	39%
Private and public hospital	14,058	3.4%
Private hospital only	25,679	6.1%
**Duration of mental health care**	-	-
Less than two years	311,943	74%
Two or more years	106,972	26%
**Severe or persistent mental illness group**	-	-
Severe or persistent mental illness	138,422	33%
Other	280,493	67%

SUPPLEMENTARY TABLE ~~1~~2. Data for Figure 1. Vaccine-preventable Hospitalisations (VPH) in NSW adults aged 18-100, by age group, gender and vaccine-preventable condition group. Rate per 100,000 person years and adjusted Incidence Rate Ratio (aIRR) after standardisation for socioeconomic disadvantage, comparing mental health service users (MH) to other NSW residents (No MH).

	**All VPH (Rate, aIRR)**			**Hepatitis (Rate, aIRR)**			**Respiratory (Rate, aIRR)**			**Other (Rate, aIRR)**		
	MH	No MH	aIRR (95%CI)	MH	No MH	aIRR (95%CI)	MH	No MH	aIRR (95%CI)	MH	No MH	aIRR (95%CI)
Females												
18-24	172	58	3.1 (2.5-3.7)	27	8	3.2 (2.3-4.6)	131	40	3.3 (2.8-3.9)	14	10	1.4 (0.9-2.2)
25-34	296	143	3.9 (3.5-4.4)	111	68	1.6 (1.4-1.9)	164	59	2.8 (2.4-3.2)	27	17	1.6 (1.2-2.2)
35-44	383	173	6.0 (5.6-6.5)	205	97	2.1 (1.9-2.4)	152	62	2.5 (2.1-2.8)	28	16	1.7 (1.2-2.3)
45-54	699	161	6.2 (5.8-6.6)	352	78	4.5 (4.1-5.0)	301	68	4.5 (4.0-5.0)	56	17	3.2 (2.5-4.2)
55-64	1,092	273	5.9 (5.6-6.2)	488	111	4.4 (3.9-4.9)	496	129	3.8 (3.5-4.3)	127	36	3.5 (2.8-4.3)
65-74	1,212	423	3.1 (2.9-3.4)	306	113	2.7 (2.3-3.2)	732	251	2.9 (2.6-3.2)	182	64	2.8 (2.3-3.5)
75-84	1,467	812	2.0 (1.8-2.2)	124	105	1.2 (0.9-1.6)	1,078	557	1.9 (1.7-2.1)	282	158	1.8 (1.5-2.2)
85+	1,824	1,558	1.2 (1.0-1.3)	35	105	0.3 (0.2-0.7)	1,418	1,123	1.3 (1.1-1.4)	372	343	1.1 (0.9-1.3)
Males												
18-24	113	37	3.2 (2.3-4.6)	23	2	10.1 (6.1-16.6)	80	25	3.2 (2.5-4.0)	10	9	1.1 (0.6-2.0)
25-34	236	60	1.6 (1.4-1.9)	90	15	5.9 (4.9-7.2)	120	33	3.7 (3.1-4.3)	25	14	1.8 (1.3-2.6)
35-44	618	103	2.1 (1.9-2.4)	415	41	10.1 (9.2-11.2)	177	47	3.8 (3.3-4.3)	41	18	2.3 (1.8-3.0)
45-54	1,084	175	4.5 (4.1-5.0)	757	91	8.3 (7.7-8.9)	301	70	4.3 (3.8-4.8)	57	17	3.4 (2.6-4.3)
55-64	2,071	351	4.4 (3.9-4.9)	1,419	194	7.3 (6.8-7.8)	608	131	4.7 (4.2-5.1)	87	33	2.7 (2.1-3.4)
65-74	1,683	539	2.7 (2.3-3.2)	623	217	2.9 (2.6-3.2)	881	264	3.3 (3.0-3.7)	200	71	2.8 (2.3-3.5)
75-84	2,025	1,019	1.2 (0.9-1.6)	323	219	1.5 (1.2-1.8)	1,423	661	2.2 (1.9-2.4)	311	154	2.0 (1.6-2.5)
85+	2,244	1,937	0.3 (0.2-0.7)	297	162	1.8 (1.3-2.6)	1,598	1,436	1.1 (1.0-1.3)	382	365	1.0 (0.8-1.4)

SUPPLEMENTARY TABLE ~~2~~3: Vaccine-preventable hospital bed days for adults (aged 18-100) to NSW public and private hospitals, July 2015 to June 2020, comparing people receiving any hospital or community mental health care to the rest of the NSW population. Adjusted incidence rate ratios (aIRR) after standardisation by (1) age and sex, (2) age, sex and socioeconomic disadvantage. Individual VPH types with fewer than 20 occurrences in the mental health group are not shown, but admissions are included in group and state totals.

	VPH bed days (n)		Crude rate (per 100k person years)			Adjusted rate ratios (aIRR), (95% CI)	
	MH care	No MH care	MH care	No MH care	IRR	1. Age, Sex.	2. Age, Sex, Disadv.
**Total Vaccine-preventable**	**80,915**	**555,863**	**5,507**	**1,894**	**2.9**	**3.1 (3.0 - 3.1)**	**3.8 (3.8 - 3.8)**
**Hepatitis B**	**29,809**	**128,404**	**2,029**	**438**	**4.6**	**4.2 (4.1 - 4.4)**	**5.7 (5.6 - 5.8)**
**Respiratory infections (1)**	**41,395**	**328,155**	**2,817**	**1,118**	**2.5**	**2.7 (2.6 - 2.7)**	**3.4 (3.4 - 3.4)**
Influenza	29,149	237,049	1,984	808	2.5	2.5 (2.5 - 2.6)	3.4 (3.4 - 3.4)
Haemophilus pneumonia	4,967	41,222	338	141	2.4	2.7 (2.5 - 3.0)	3.1 (3.0 - 3.2)
Streptococcus pneumoniae	7,379	46,041	502	157	3.2	3.5 (3.2 - 3.7)	4.0 (3.9 - 4.1)
COVID	352	7,788	24	27	0.9	1.0 (0.6 - 1.4)	1.0 (0.9 - 1.1)
Pertussis (whooping cough)	426	2,904	29	10	2.9	3.0 (2.2 - 4.0)	4.4 (3.9 - 4.9)
**Other (2)**	**11,004**	**107,547**	**749**	**367**	**2.0**	**2.1 (2.0 - 2.2)**	**2.8 (2.7 - 2.8)**
Herpes Zoster	6,982	69,075	475	235	2.0	2.2 (2.0 - 2.4)	2.9 (2.8 - 2.9)
Tuberculosis	3,006	30,435	205	104	2.0	1.6 (1.4 - 1.9)	2.4 (2.3 - 2.5)
Varicella (Chicken Pox)	881	6,622	60	23	2.7	2.2 (1.8 - 2.7)	3.4 (3.1 - 3.6)
Rotaviral enteritis	386	2,575	26	9	3.0	2.4 (1.7 - 3.3)	4.1 (3.7 - 4.6)

Notes: VPH: Vaccine-preventable Hospitalisation. MH: mental health. Disadv: Disadvantage, measured using index of relative socioeconomic disadvantage (IRSD) of address of residence. (1) Respiratory group total includes 9 hospitalisations with Diptheria. (2) Other group total includes 22 hospitalisations with Mumps, Measles, Haemophilus meningitis, Tetanus, Rubella, Cholera, Acute poliomyelitis. The sum of individual conditions may exceed group totals because some admissions have more than one VPH diagnosis recorded.

SUPPLEMENTARY TABLE S34: Subgroup analysis of Vaccine-preventable hospitalisations: Rate per 100,000 person years and adjusted Incidence Rate Ratio (aIRR) for mental health service users overall, and divided into subgroups of (i) severe and persistent mental illness or (ii) other mental health service users. Severe and persistent mental illness defined as any diagnosis of psychosis, bipolar disorder or psychotic depression, or more than two years of mental health service use. Admissions from July 2015 to June 2020. Standardised rates not calculated where there were 20 or fewer admissions in the period in the mental health group.

	Rest of NSW Population		All MH service users (n = 418,915)			Severe and Persistent Mental Illness (n = 138,422)		Other Mental Health service users (n=280,493)	
	PPH events	Rate	PPH events	Rate	aIRR (95% CI)	Rate	aIRR (95% CI)	Rate	aIRR (95% CI)
**Total Vaccine-preventable**	83,501	285	10,679	727	3.2 (3.1 - 3.3)	1,052	4.0 (3.9 - 4.1)	783	2.8 (2.8-2.9)
**Hepatitis B**	26,371	90	4,901	334	4.4 (4.3 - 4.6)	501	6.1 (5.8 - 6.3)	309	3.6 (3.4-3.7)
**Respiratory infections**	45,820	156	4,918	335	2.8 (2.7 - 2.9)	488	3.4 (3.2 - 3.6)	387	2.6 (2.5-2.7)
Influenza	35,381	121	3,609	246	2.7 (2.6 - 2.8)	357	3.2 (3.0 - 3.4)	290	2.5 (2.4-2.6)
Haemophilus pneumonia	4,618	16	505	34	2.8 (2.6 - 3.1)	52	3.6 (3.2 - 4.2)	38	2.5 (2.2-2.8)
Streptococcus pneumonia	6,220	21	901	61	3.6 (3.3 - 3.9)	90	4.7 (4.2 - 5.2)	64	3.1 (2.8-3.5)
COVID	641	2.2	27	1.8	0.9 (0.6 - 1.3)	-	-	-	-
Pertussis	449	1.5	55	3.7	3.1 (2.3 - 4.2)	4.5	3.2 (2.0 - 5.2)	5.0	3.4 (2.4-4.9)
**Other**	12,535	43	1,029	70	2.2 (2.0 - 2.3)	80	2.0 (1.8 - 2.3)	101	2.4 (2.2-2.6)
Herpes Zoster	9,106	31	761	52	2.3 (2.1 - 2.5)	59	2.1 (1.8 - 2.4)	77	2.6 (2.3-2.8)
Tuberculosis	2,301	7.8	162	11.0	1.8 (1.5 - 2.1)	13	1.8 (1.3 - 2.4)	15	2.0 (1.6-2.5)
Varicella (Chicken Pox)	1,108	3.8	101	6.9	2.1 (1.7 - 2.7)	6.6	1.9 (1.3 - 2.8)	9.0	2.5 (1.9-3.2)
Rotaviral enteritis	401	1.4	39	2.7	2.5 (1.7 - 3.5)	-	-	-	-

SUPPLEMENTARY TABLE S5: Subgroup analysis of Vaccine-preventable hospitalisations: Rate per 100,000 person years and adjusted Incidence Rate Ratio (aIRR) for mental health service users overall, and divided into subgroups of (i) people with at least one public or private hospital admission for mental health care or (ii) people whose only mental health care occurred in community settings. Admissions from July 2015 to June 2020. Standardised rates not calculated where there were 20 or fewer admissions in the period in the mental health group.

	Rest of NSW Population		All MH service users			Any mental health hospitalisation		Community mental health care only	
	PPH events	Rate	PPH events	Rate	aIRR (95% CI)	Rate	aIRR (95% CI)	Rate	aIRR (95% CI)
**Total Vaccine Preventable**	83,501	285	10,679	727	3.2 (3.1 - 3.3)	806	3.6 (3.5 -3.7)	676	3.1 (3.0 -3.2)
**Hepatitis B**	26,371	90	4,901	334	4.4 (4.3 - 4.6)	407	5.3 (5.1 -5.5)	267	3.9 (3.8 -4.2)
**Respiratory infections**	45,820	156	4,918	335	2.8 (2.7 - 2.9)	349	3.1 (2.9 -3.2)	338	2.8 (2.7 -2.9)
Influenza	35,381	121	3,609	246	2.7 (2.6 - 2.8)	249	2.9 (2.7 -3.0)	259	2.7 (2.6 -2.9)
Haemophilus pneumonia	4,618	16	505	34	2.8 (2.6 - 3.1)	39	3.4 (3.0 -3.9)	31	2.5 (2.1 -2.9)
Streptococcus pneumonia	6,220	21	901	61	3.6 (3.3 - 3.9)	67	4.1 (3.7 -4.5)	56	3.5 (3.1 -3.9)
COVID	641	2.2	27	1.8	0.9 (0.6 - 1.3)	-	-	-	-
Pertussis	449	1.5	55	3.7	3.1 (2.3 - 4.2)	3.4	3.4 (2.2 -5.2)	4.4	3.1 (2.1 -4.7)
**Other**	12,535	43	1,029	70	2.2 (2.0 - 2.3)	61	2.0 (1.8 -2.2)	83	2.7 (2.4 -2.9)
Herpes Zoster	9,106	31	761	52	2.3 (2.1 - 2.5)	44	2.1 (1.9 -2.3)	63	2.8 (2.5 -3.1)
Tuberculosis	2,301	7.8	162	11.0	1.8 (1.5 - 2.1)	10	1.5 (1.1 -1.9)	12	2.3 (1.9 -2.9)
Varicella (Chicken Pox)	1,108	3.8	101	6.9	2.1 (1.7 - 2.7)	5.5	1.8 (1.3 -2.5)	8.5	2.9 (2.2 -3.8)
Rotaviral enteritis	401	1.4	39	2.7	2.5 (1.7 - 3.5)	-	-	-	-
